# Deciphering Dickerson–Drew DNA Equilibrium beyond the BI/BII DNA Dichotomy by Interpretation of ^31^P NMR Parameters

**DOI:** 10.1021/acs.jctc.5c01076

**Published:** 2025-09-16

**Authors:** Jiří Fukal, Miloš Buděšínský, Jakub Šebera, Marie Zgarbová, Petr Jurečka, Vladimír Sychrovský

**Affiliations:** † Institute of Organic Chemistry and Biochemistry, Czech Academy of Sciences, 166 10 Praha 6, Czech Republic; ‡ Department of Physical Chemistry, Faculty of Science, 48207 Palacký University Olomouc, 77146 Olomouc, Czech Republic; § J. Heyrovský Institute of Physical Chemistry, Czech Academy of Sciences, 182 00 Prague 8, Czech Republic

## Abstract

DNA duplexes exist as dynamic ensembles of interconverting conformations in solution. Conventional nuclear magnetic resonance (NMR) data interpretation often simplifies this behavior by assuming one dominant structure, but multiple substates (such as different backbone conformers) can coexist. Here, we present an approach that refines the interpretation of ^31^P NMR data in the Dickerson–Drew DNA by integrating a nucleotide conformational classification (NtC) (Černý et al., *Nucleic Acids Research* 2020, **48**, 6367–6381) with molecular dynamics (MD) simulations. By finely classifying backbone conformers into distinct NtC-defined states and using MD to predict their populations, we achieve a more nuanced correspondence between experimental NMR observables and DNA structure-dynamical heterogeneity. Application of this framework demonstrates a radical improvement of NMR data interpretation, thereby enhancing the reliability of deducing DNA conformational equilibria in solution.

## Graphical abstract

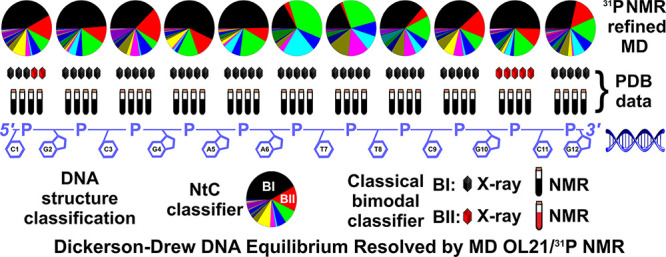

## Introduction

DNA is not a rigid molecule; even within the classical B-form double helix, local geometries fluctuate among substates. Nucleic acid structural dynamics is fundamental to their biological functions.^1–4^ The DNA backbone phosphates can involve multiple torsional orientations (commonly denoted BI and BII),^5^ often utilized as a basis for interpreting NMR and other physicochemical data.^6–8^ However, NMR spectroscopy is a powerful technique to probe DNA conformational dynamics in solution in more detail. Interpreting the spectral measurements with a simple two-state model or even with a single static structure can be misleading when a distribution of states is actually present. A commonly held assumption that DNA exists in a homogeneous state, recognized as an equilibrium between BI and BII conformations, is revised due to the relevant interpretation of experimental NMR data in this work.

The analysis of structural data from the Protein Data Bank (PDB) has revealed a wider array of conformational states,^9^ enabling more nuanced discrimination of nucleic acid geometries beyond the classical BI/BII classification. To systematically categorize DNA conformations,^10,11^ an NtC (diNucleotide Conformer) classification has been developed based on a comprehensive survey of dinucleotide structures.^9,12^ In the relevant analysis, over 60,000 dinucleotide steps from high-resolution crystal structures were grouped into 44 discrete conformational classes termed NtC. Each NtC class represents a distinct combination of backbone torsion angles and sugar pucker, effectively creating a “DNA structural alphabet”. Any nucleic acid structure can be readily classified using the DNATCO web server by uploading its corresponding PDB file.^13,14^ An automated way to assign a given DNA structure (experimental or simulated) to the nearest NtC conformational class enabled interpretation of ^31^P NMR parameters in the Dickerson–Drew DNA (Figure 1A,B) beyond the classical BI (ε – ζ < 0°)/BII (ε – ζ ≥ 0°) dichotomy.

**Figure 1:**
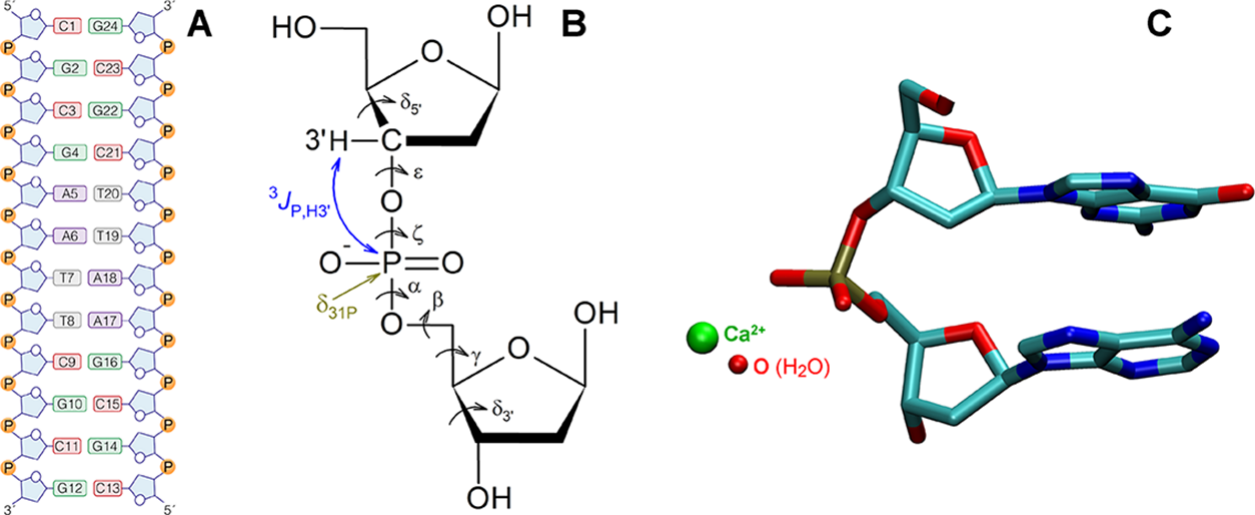
Dickerson–Drew DNA. (A) Schematic representation of nucleotide sequence (A—adenine, T—thymine, C—cytosine, G—guanine, P—phosphate group). (B) Sugar–phosphate-sugar fragment (sPs) with backbone torsion angles δ_5′_, ε, ζ, α, β, γ, δ_3′_. (C) Solvation of G4pA5 phosphate in the crystal structure with PDB ID 463D.

Molecular dynamics (MD) simulations offer a complementary approach to exploring DNA flexibility by generating an ensemble of structures over time. The distribution of DNA conformational states, recognized as a DNA equilibrium, depends on the given nucleotide sequence, solvent composition, temperature, and other physicochemical conditions.^15–21^ Characterization of these states^22,23^ is commonly assisted by MD simulations, often constrained by experimental NMR data^24^ interpreted as structural constraints.^25^ Indeed, many NMR-derived structures in the PDB were resolved through this approach.^26^ Given the critical impact of NMR data interpretation on structural resolution, quantum chemical (QM) methods,^27,28^ including MD^29,30^ and hydration/solvation,^31–33^ can be employed to improve usual (empirical) interpretation schemes. However, QM-MD approaches to interpret NMR data remain relatively rare due to their computational demands.^34,35^ The small variation in ^31^P NMR parameters (Figure 1B) across different DNA conformations^36–43^ imposes stringent accuracy requirements on experimental data interpretation. In this study, we employ the QM-MD method^44,45^ previously benchmarked for rapid and accurate averaging of ^31^P NMR parameters.^46^ NMR spectroscopy is uniquely suited to revealing three-dimensional nucleic acid structures in solution.^47–52^ We apply an NtC-based analysis to MD simulation ensembles and demonstrate how this framework improves the assignment of conformational equilibria of Dickerson–Drew DNA from ^31^P NMR measurements. We show that such an integrated approach yields deeper insight into DNA’s solution structure than traditional methods, aligning the analysis with the inherent polymorphism of the DNA helix. Last but not least, this approach allows robust interpretation of experimental data and provides a foundation for the development and validation of force fields that are consistent with experimental NMR data.^53,54^

## Materials and Methods

### DNA Samples and NMR Measurements

The Dickerson–Drew DNA duplex, comprising the self-complementary palindromic oligonucleotides 1:5′-CGCGAATTCGCG-3′ and 2:5′-CGCGAATTCGCG-3′ (Figure 1A), synthesized by Generi Biotech (Czech Republic), was dissolved in a 9:1 H_2_O:D_2_O mixture.^31^P chemical shifts (δ_31P_) were measured on a Bruker AVANCE-500 instrument (operating at 202.3 MHz for ^31^P) equipped with a 5 mm TBO BB-probe. External referencing was performed with an in-capillary H_3_PO_4_ standard, and the measured accuracy was about 0.01 ppm. δ_31P_ shifts dependence on temperature ranging from 10 to 35 °C was measured by the proton broadband-decoupled ^31^P NMR spectra with a 5 °C step.

### MD Simulations

MD simulations were initiated from the DNA structure with PDB ID 1NAJ^55^ solvated in an octahedral box of 12 Å of SPC/E water.^56^ Potassium and chloride ions, Joung and Cheatham parameters,^57,58^ were added to neutralize the system and maintain a 0.15 M KCl concentration. The system was stepwise relaxed by minimization of H atoms with other atoms restrained (1000 kcal/(mol·Å^2^)), minimization of solvent and ions with solute atoms restrained (1000 kcal/(mol·Å^2^)), a short NpT MD of solvent and ions with solute atoms restrained (500 kcal/(mol·Å^2^)) upon gradual heating from 10 to 293 K, minimizations with progressively decreasing restraints on solute’s heavy atoms (1000, 500, 125, 25, and 0 kcal/(mol·Å^2^)), unrestrained NpT MD upon gradual heating from 10 to 293 K in 100 ps and a final 50 ps NpT MD at 300 K. Production NpT MD simulation (2 μs) was performed using CUDA PMEMD code in the AMBER 18 package^59^ at 1 bar and 293 K with Monte Carlo barostat (taup = 2) and Langevin thermostat (collision freq 5 ps^–1^), SHAKE on bonds to H atoms and 10 Å direct space nonbonded cutoff. MD simulations at temperatures 10, 15, 20, 25, 30, and 35 °C were conducted under the same conditions on a 2 μs time scale. Fraying of DNA end-base pairs^60^ was diminished by a mild flat well restraints discriminated by a distance between the Watson–Crick-bonded electronegative atoms: flat potential (2.5 ÷ 3.2 Å), below 2.5 Å (parabolic restraint, *k* = 20 kcal/(mol·Å^2^)), above 3.2 Å (parabolic restraint, *k* = 30 kcal/(mol·Å^2^)). MD snapshots (10 ps frame) were analyzed using the cpptraj (AMBER) and the nastruct and multidihedral tools and NtC classified,^14^ as described below. The OL21 ^61^ and bsc1 ^62^ force fields were employed.

### QM NMR Calculations

Geometry optimizations of sPs DNA fragments (Figure 1B) were performed using the B3LYP functional,^63–66^ the 6-31++G­(d) basis set,^67–71^ and SMD water solvation,^72^ with “verytight” SCF convergence and the integration grid 199974. Backbone torsion angles (Figure 1B) were fixed according to the values defining each NtC class (Table S6).^9,12^ ^3^ *J*_P,H3′_ couplings were computed using the CP DFT method^73,74^ with the pcJ-3 basis,^75^ accounting for all four scalar coupling contributions. Chemical shieldings σ_31P_ were calculated by the GIAO approach^76–78^ with the IGLO-III basis set.^79^ The reliability of these QM methods has been validated previously in benchmarked studies aimed at performances of different DFT and QM methods, including spin–orbit interaction, various atomic bases, and solvent description.^46,80^

### NtC Classification

MD snapshot geometries were assigned to NtC classes based on minimal RMSD in backbone torsion space (*t* ∈ (δ_*n*_, χ_*n*_, ε_*n*_, ζ_*n*_, α_*n*+1_, β_*n*+1_, γ_*n*+1_, δ_*n*+1,_ χ_*n*+1_) compared to class-defining values (Table S6).^9,12^

$$\sqrt{\sum_{t}(t_{\mathrm{NtC}}-t_{\mathrm{MD}}{)}^{2}}$$ 1

Experimental DNA structures in the PDB repository^26^ were NtC classified using the DNATCO web server,^13,14^ accessible at https://dnatco.datmos.org.

### Calculation of NMR Observables from MD

Population weighting was performed using population weights

$$w_{\mathrm{NtC}}=\frac{1}{N}\overset{N}{\underset{i=1}{\sum }}\omega_{i}=\frac{N^{\mathrm{NtC}}}{N};\;\omega_{i}=1\;\mathrm{for}\;i\in \mathrm{NtC},\omega_{i}=0\;\mathrm{otherwise}$$ 2

$$\sum_{i\in \mathrm{NtC}}w_{i}=1$$ 3

calculated as relative occurrences of NtC-classified MD snapshots (eq 1) in N MD snapshots. Dynamically averaged values of σ_31P_ shielding and ^3^ *J*_PH3′_ coupling

$$\sigma =\sum_{i\in \mathrm{NtC}}w_{i}\sigma_{i}$$ 4

$$J=\sum_{i\in \mathrm{NtC}}w_{i}J_{i}$$ 5

were calculated as population-weighted averages of class-specific spectral parameter values (Table S6).

Probability averaging^44,45^ extends the population weighting approach by incorporating parameters’ geometrical dependences (Figures S7 and S8), averaged by relevant probability distributions (Figures S9 and S10). The class-specific parameter values in eqs 4 and 5 are thus substituted by their probability average values due to torsional motions in DNA phosphate. Probability distribution of a torsion angle *t*

$$P^{\mathrm{NtC}}(t_{k})=\frac{1}{N^{\mathrm{NtC}}}\sum_{i\in \mathrm{NtC}}\omega_{i};,\omega_{i}=1\;\mathrm{for}\;t_{i}\in \langle 10^{{}^{\circ}}(k-1),10^{{}^{\circ}}k),\;\omega_{i}=0\;\mathrm{otherwise}$$ 6

$$P^{\mathrm{NtC}}(t_{k})≡\{P(t_{k})\};\;k=1,2,\cdot \cdot \cdot ,36$$ 7

$$\sum_{k}P^{\mathrm{NtC}}(t_{k})=1$$ 8

is calculated as normalized occurrence of *t* values in NtC-classified MD snapshots discretized with a 10° step. Probability averaging of spectral parameters

$$\sigma =\sum_{i\in \mathrm{NtC}}w_{i}\sigma_{i}=\sum_{i\in \mathrm{NtC}}w_{i}\sum_{\left(k,j)=(1,1\right)}^{\left(36,36\right)}P^{i}(\alpha_{k},\zeta_{j})\sigma^{i}(\alpha_{k},\zeta_{j})$$ 9

$$J=\sum_{i\in \mathrm{NtC}}w_{i}J_{i}=\sum_{i\in \mathrm{NtC}}w_{i}\sum_{k=1}^{36}P^{i}(\varepsilon_{k})J^{i}(\varepsilon_{k})$$ 10

included contributions from the BI family (BB00 and BB01) and the BII family (BB04 and BB07) NtC states; other states were treated by population averaging.

^31^P chemical shift referencing employed the A6pT7 phosphate as a DNA-internal standard.

In theory, the ^31^P chemical shift of DNA phosphate was calculated as a difference of chemical shielding constants QM calculated in relevant phosphates.

δ=σ(A6pT7)−σ 11

In the experiment, the ^31^P chemical shift of DNA phosphate was referenced to the chemical shift of A6pT7 phosphate

$$\delta =\delta^{’}-\delta^{’}\left(A6\mathrm{pT}7\right)$$ 12

where δ’ and δ’(A6pT7) shifts were originally measured relative to an external standard (here H_3_PO_4_). Such coherent referencing assured unbiased comparison of theoretical and experimental chemical shifts and mitigated potential unwanted effects due to inadequate theoretical H_3_PO_4_ reference.^81^ Accuracy of spectral data interpretation was evaluated by mean absolute deviation (MAD)

$$\mathrm{MAD}=\sqrt{\frac{1}{N}\sum_{i=1}^{N}(X_{\mathrm{calc},i}-X_{\exp,i}{)}^{2}},X=J,\delta$$ 13

of *N* parameters from the experiment.

Effect on NMR parameters due to phosphate solvation/Mg^2+^ was QM calculated for selected NtC phosphates derived from the DNA crystal structure 463D (Figure 1C).

The missing water molecules were added manually to maintain octahedral ion coordination, and the geometry of the complex was subsequently QM optimized. The NMR parameter values (Table S11) in geometry-optimized, solvated BB00, BB01, BB04, and BB07 phosphates (Figure S11) were used in population-weighted averaging (eqs 4 and 5).

## Results

### NMR Experimental Data

The δ_31P_ chemical shifts obtained in this study, as well as those reported in the literature^82–84^ (Table S1), varied due to differences in referencing. After the unified referencing (eq 12, Methods), the chemical shifts were found to differ by no more than 0.14 ppm (Figure 2A, Table S2). The total range of δ_31P_ shifts (Figure 2C) decreased with temperature, from 0.57 ppm at 10 °C to 0.49 ppm at 35 °C (Table S3).

**Figure 2:**
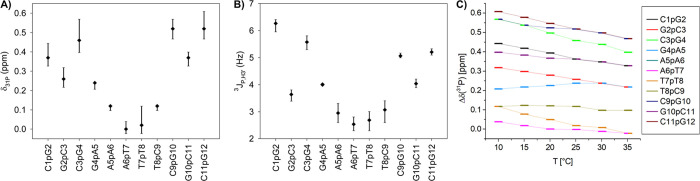
Experimental ^31^P NMR parameters. (A) ^31^P chemical shifts. (B) ^3^ *J*_PH3’_ scalar couplings. (C) Dependence of ^31^P chemical shifts on temperature referenced to the shift value at 20 °C (Table S4); Δδ_31P_ = δ_31P_ (*T*) −δ_31P_ (20 °C). The δ_31P_ shifts (◆) in this work (Table 1) with spectral parameter ranges due to temperature variation. Average ^3^ *J*_PH3’_ couplings (◆) in the literature (Tables 2 and S5) with spectral parameters ranges in different experiments.

The ^3^ *J*_PH3’_ coupling constants (Figure 2B) showed up to 0.8 Hz variation across data sets^83,85,86^ (Table S5). Nevertheless, these studies report comparable experimental accuracies,^86^ and we, therefore, used average ^3^ *J*_PH3’_ values (Table 2) for subsequent analysis.

### NMR Parameter Assignment in Experimental DNA Structures

The assignment of spectral parameters in experimental DNA structures was aided by NtC classification of backbone geometries using the DNATCO web tool (Methods) and by QM calculations for NtC-specific phosphate models (Table S6). The chemical shifts in crystal structures generally fell within the experimental range with some overestimation observed in DNA tail-region phosphates (Table 1, Figure S1). Conversely, most ^3^ *J*_PH3’_ couplings were underestimated, except for the 10p11 phosphate (Table 2, Figure S2A).

**Table 1:** δ_31P_ Shifts (ppm) Calculated for X-ray and NMR Experimental DNA Structures and Measured in DNA

	calculation					
	X-ray structure^a^		NMR structure^a^		experiment^b^	
DNA	average	range	average	range	this work	range
C1pG2	1.05	0.07 ÷ 2.18	0.10	0.06 ÷ 0.14	0.37	0.33 ÷ 0.40
G2pC3	0.75	0.04 ÷ 1.69	0.02	0.00 ÷ 0.06	0.26	0.20 ÷ 0.28
C3pG4	0.24	0.23 ÷ 0.27	0.10	0.06 ÷ 0.14	0.46	0.36 ÷ 0.50
G4pA5	0.21	0.06 ÷ 0.42	0.10	0.06 ÷ 0.14	0.24	0.22 ÷ 0.30
A5pA6	–0.03	–0.08 ÷ 0.03	0.09	0.02 ÷ 0.14	0.12	0.10 ÷ 0.14
A6pT7	0.00	0.00	0.00	0.00	0	0.00
T7pT8	0.06	–0.08 ÷ 0.53	–0.02	–0.08 ÷ 0.07	0.02	0.00 ÷ 0.03
T8pC9	0.00	–0.08 ÷ 0.11	0.03	0.00 ÷ 0.06	0.12	0.12 ÷ 0.17
C9pG10	0.11	0.06 ÷ 0.19	0.10	0.06 ÷ 0.14	0.52	0.47 ÷ 0.54
G10pC11	4.10	3.54 ÷ 4.56	0.01	–0.08 ÷ 0.06	0.37	0.33 ÷ 0.38
C11pG12	0.49	0.00 ÷ 1.19	0.00	–0.08 ÷ 0.06	0.52	0.47 ÷ 0.55
MAD^c^	1.16	0.99 ÷ 1.47	0.27	0.24 ÷ 0.32	–	–

a Theoretical average values and ranges in DNA (Table S9).

b This work (phosphate buffer, pH 7.0, EDTA, NaCl, 20 °C) and ranges in other experiments (Table S2).

c The mean absolute deviation. The δ_31P_ shifts referenced to A6pT7 phosphate.

**Table 2:** ^3^ *J*_PH3’_ Couplings (Hz) Calculated for X-ray and NMR Experimental DNA Structures and Measured in DNA

	calculation				
	X-ray structure^a^		NMR structure^a^		
DNA	average	range	average	range	experiment^b^
C1pG2	4.49	1.94 ÷ 6.39	1.92	1.92	6.27
G2pC3	3.43	1.95 ÷ 5.37	1.66	1.45 ÷ 1.92	3.64
C3pG4	2.52	2.52	1.91	1.87 ÷ 1.92	5.58
G4pA5	2.70	1.92 ÷ 3.86	1.92	1.92	4.00
A5pA6	1.59	1.45 ÷ 1.70	1.89	1.78 ÷ 1.92	2.95
A6pT7	1.85	1.70 ÷ 1.95	1.70	1.45 ÷ 1.95	2.53
T7pT8	2.00	1.45 ÷ 4.20	1.51	1.45 ÷ 1.68	2.69
T8pC9	1.70	1.45 ÷ 2.23	1.68	1.45 ÷ 1.92	3.07
C9pG10	2.04	1.92 ÷ 2.22	1.91	1.87 ÷ 1.92	5.07
G10pC11	10.06	9.26 ÷ 10.86	1.64	1.45 ÷ 1.92	4.04
C11pG12	3.64	1.70 ÷ 5.24	1.57	1.45 ÷ 1.92	5.21
MAD^c^	2.46	2.54 ÷ 2.46	2.61	2.50 ÷ 2.32	–

a Theoretical average values and ranges in DNA (Table S10).

b Average ^3^ *J*_P,H3’_ coupling, as measured by Clore,^85^ Sklenář^86^ and Wu^83^ (Table S5).

c Mean absolute deviation.

The NtC classes found in crystal structures included BB00 (canonical BI), BB01 and BA05 (BI representatives), BB04 (BI – BII bridging class according to ζ torsion), BA01 (BI – BII bridging class according to β torsion), and BB07 (canonical BII). BI-type phosphates (BB00, BB01) predominated in the DNA stem, whereas BII-type phosphates (BB07) were more prevalent in the DNA tail regions. Minor occupations of other NtC phosphates, such as AB01, BB01, and BA05 (Table S7), were also found in crystal structures.

In contrast, NMR structures displayed a narrower range of spectral parameters and were overwhelmingly composed of BI-type phosphates (BB00, BB01, and BA05) (Table S8). The MAD of the ^31^P shifts from the experiment was 0.27 ppm for NMR structures and 1.16 ppm for crystal structures (Table 1). The corresponding MADs for ^3^ *J*_PH3’_ couplings were 2.61 and 2.46 Hz, respectively (Table 2).

The systematic differences between X-ray and NMR structures were evident from the assigned NMR parameters (Figure S6). Structural variability was markedly higher in crystal DNA tail phosphates compared with their NMR counterparts. Importantly, the BB07 class (canonical BII) showed overestimated values of spectral parameters (Figures S4 and S5, Table S7), while the uniform BI-like phosphates led to underestimated spectral parameters (Figures S1 and S3). This suggests that properly balanced populations of NtC phosphates like those found in crystal structures would improve the agreement of assigned spectral parameters with the experiment.

NtC classification allowed for a more detailed resolution of backbone geometries than the classical BI/BII dichotomy. BI–BII intermediate phosphates, such as BB04 and BA01, with ^31^P shifts of 0.64 and 0.52 ppm, respectively, enhanced the resolution of backbone geometries compared to canonical BI (BB00) and BII (BB07) phosphates, which differ by 4.23 ppm. It should be noted that the BI (ε – ζ < 0°)/BII (ε – ζ ≥ 0°) DNA classification relies solely on ε and ζ torsion angles, whereas NtC classes incorporate all backbone torsions (Figure 1B). Nevertheless, the overall populations of canonical BI (BB00) and BII (BB07) phosphates, as determined by MD in DNA, are considerable (Table S6).

### QM NMR Calculations of Solvated Representative NtC Phosphates

The influence of phosphate solvation/Mg^2+^ on the spectral parameters was evaluated for the BB00, BB01, BB04, and BB07 NtC phosphates (Figure S11). Upon solvation, the ^31^P shielding constant, σ_31P_, increased most notably by 2.49 ppm for the BB07 phosphate and decreased by 0.10 ppm for the BB04 phosphate (Table S11). Similar Mg^2+^-induced shielding effects have been reported previously upon MD-averaged metal ion coordination in B-DNA.^41^ Specifically, the δ_31P_ shift of the BB07 (canonical BII) phosphate decreased from 4.23 to 2.55 ppm upon solvation/Mg^2+^. For BB04 (a BI–BII bridging class), the δ_31P_ shift increased from 0.64 to 1.55 ppm. These substantial effects on ^31^P shifts, especially for BII-like states, suggest that explicit solvation/Mg^2+^ is essential for accurate chemical shift prediction in structurally different NtC phosphates. In contrast, changes in ^3^ *J*_PH3’_ couplings due to solvation were relatively minor, especially when considering the parameter variations in different experiments (Table S5). Maximal effects on ^3^ *J*_PH3’_ couplings ranged between −0.18 Hz for BB01 and +0.21 Hz for the BB07 phosphate.

### Correlation of MD with NMR Observables

Population-weighted averaging (Tables 3 and 4) demonstrated that NtC populations significantly influence NMR parameters (Figures 3C and S12). More advanced probability averaging led to modest changes in assigned spectral parameters. The maximal effect on ^31^P shift was +0.24 ppm in the 1p2 DNA phosphate and –0.82 Hz on ^3^ *J*_PH3’_ coupling in the 4p5 phosphate. These DNA phosphates exhibited significant BB07 populations (Figure S12) and large-amplitude torsional fluctuations (Figures S9 and S10), highlighting the value of probability averaging due to parameter geometrical dependences (Figures S7 and S8).

**Figure 3:**
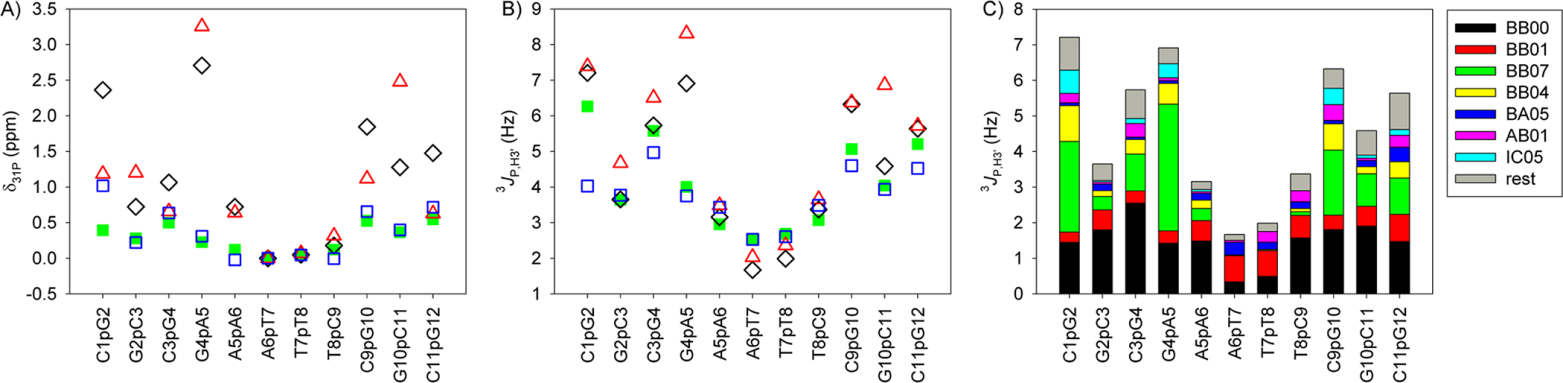
^31^P NMR parameters. (A) δ_31P_ chemical shifts. (B) ^3^ *J*_P,H3’_ scalar couplings. (C) ^3^ *J*_P,H3’_ coupling contributions due to NtC phosphates. Calculations were made by probability averaging with the OL21 (black diamond) and bsc1 (red triangle) force fields, with the OL21 force field including phosphate solvation/Mg^2+^ and fitted NtC populations to experimental data (blue square). The measured spectral parameters (solid green square).

**Table 3:** δ_31P_ Shifts (ppm) in DNA

DNA	OL21^a^	OL21^b^	OL21^c^	OL21^d^	OL21^e^	bsc1^a^	bsc1^b^	experiment^f^
C1pG2	2.12	2.36	1.53	1.50	1.02	0.94	1.18	0.37
G2pC3	0.73	0.73	0.44	0.50	0.22	1.13	1.20	0.26
C3pG4	1.02	1.07	0.63	1.12	0.64	0.63	0.65	0.46
G4pA5	2.58	2.71	1.56	0.73	0.31	2.97	3.25	0.24
A5pA6	0.64	0.72	0.33	0.22	–0.02	0.46	0.64	0.12
A6pT7	0.00	0.00	0.00	0.00	0.00	0.00	0.00	0.00
T7pT8	0.04	0.05	0.05	0.00	0.05	0.05	0.07	0.02
T8pC9	0.22	0.18	0.11	0.13	0.00	0.29	0.31	0.12
C9pG10	1.66	1.85	1.14	1.07	0.65	0.99	1.11	0.52
G10pC11	1.29	1.28	0.84	0.72	0.40	2.39	2.47	0.37
C11pG12	1.19	1.48	0.91	1.02	0.72	0.53	0.62	0.52
MAD^g^	1.04	1.15	0.60	0.50	0.22	1.09	1.20	–

a Population weighting.

b Probability averaging.

c Population weighting including phosphate solvation.

d Population weighting with BB00, BB01, BB04, and BB07 NtC populations fitted to ^3^ *J*_P,H3’_ experiment.

e Population weighting including solvation/Mg^2+^ and fitted NtC populations.

f Experiment in this work (phosphate buffer, pH 7.0, EDTA, NaCl, 20 °C).

g Mean absolute deviation.

**Table 4:** ^3^ *J*_P,H3’_ Coupling Constants (Hz) in DNA

DNA	OL21^a^	OL21^b^	OL21^c^	OL21^d^	OL21^e^	bsc1^a^	bsc1^b^	experiment^f^
C 1pG2	7.35	7.21	7.37	3.61	4.03	6.42	7.39	6.27
G2pC3	3.79	3.65	3.72	3.06	3.77	4.61	4.67	3.64
C 3pG4	4.95	5.73	4.92	4.02	4.97	5.24	6.51	5.58
G4pA5	7.73	6.91	7.79	2.91	3.75	8.60	8.31	4.00
A5pA6	3.59	3.16	3.51	2.70	3.43	3.19	3.49	2.95
A6pT7	2.03	1.67	1.93	2.53	2.53	2.05	2.03	2.53
T7pT8	2.24	1.99	2.15	2.80	2.61	2.18	2.36	2.68
T8pC9	2.90	3.37	2.81	3.06	3.49	3.09	3.65	3.07
C9pG10	6.08	6.32	6.08	3.77	4.60	5.23	6.38	5.07
G10pC11	4.97	4.59	4.94	3.26	3.93	7.26	6.87	4.04
C11pG12	5.25	5.64	5.22	4.03	4.52	4.61	5.72	5.21
MAD^g^	1.29	1.08	1.31	1.16	0.77	1.75	1.72	–

a Population weighting.

b Probability averaging.

c Population weighting including phosphate solvation.

d Population weighting with BB00, BB01, BB04, and BB07 NtC populations fitted to the δ_31P_ experiment.

e Population weighting including solvation/Mg^2+^ and fitted NtC populations.

f Average of experimental ^3^ *J*_P,H3’_ couplings (Table S5).

g Mean absolute deviation.

Overall, MD-averaged ^31^P shifts (Figure 3A) were elevated in DNA tail phosphates compared to stem regions, in line with increased BB07 and BB04 populations. However, these calculated increases typically exceeded the experimental values. The ^3^ *J*_PH3’_ couplings (Figure 3B) were overestimated in tail phosphates and underestimated in the stem, correlating with BB00/BB01 dominance in the latter (Figure 3C).

Agreement with experiment was better for OL21 than for the bsc1 force field, as reflected in the lower MAD values for assigned parameters (Tables 3 and 4). Differences in NtC population trends between the two force fields were especially evident toward the DNA termini (Figure S13). Spectral convergence was also faster with OL21 (Figures S14–S15), although exact equivalence of parameters in NMR-equivalent sites in this palindromic DNA was not achieved. Reducing discrepancies between NMR-equivalent phosphates required at least 1 μs of MD simulation with OL21, whereas the bsc1 force field performed even worse.

Notably, the MAD values (Tables 3 and 4) decreased with MD averaging, more prominently for ^3^ *J*_PH3’_ couplings than for ^31^P shifts, confirming the benefits of incorporating DNA structural dynamics.

### Solvation Effect on MD-Averaged NMR Parameters

The relative increases of spectral parameters for DNA tail phosphates (Figure 3A,B) were in qualitative agreement with the experimental data. However, systematic deviations of both ^31^P chemical shifts and ^3^ *J*_PH3’_ couplings suggested a common deficiency in the models, likely attributable to inadequate treatment of phosphate solvation. Including solvation effects in the population-weighted averaging reduced the MAD for ^31^P shifts from 1.04 to 0.59 ppm (Table 3). For the ^3^ *J*_PH3’_ couplings, the MAD increased negligibly by 0.02 Hz (Table 4).

Neither the OL21 nor the bsc1 force fields provided NtC phosphate populations that fully matched experimental observations. This may be linked to previously reported force field limitations.^87^ Nonetheless, NtC-based classification of DNA structural dynamics enabled a qualitatively improved assignment of spectral parameters over the traditional BI/BII model pervasive in the literature.^7,82^ Adjusting NtC populations to better align with experimental data may offer a promising route for refining force field parameters.

### Refinement of Conformational Equilibria Interpretation with Experimental Data

Approximately 60–80% of all phosphates in the DNA were found to belong to four NtC classes: BB00, BB01, BB04, and BB07 (Figure 4A). These classes were selected for population fitting against the experimental data. BB00 and BB01 were grouped as BI′, and BB04 and BB07 were grouped as BII′, with population ratios within each group fixed, as calculated with the OL21 force field. All other NtC populations were retained as calculated with the OL21 force field.

**Figure 4:**
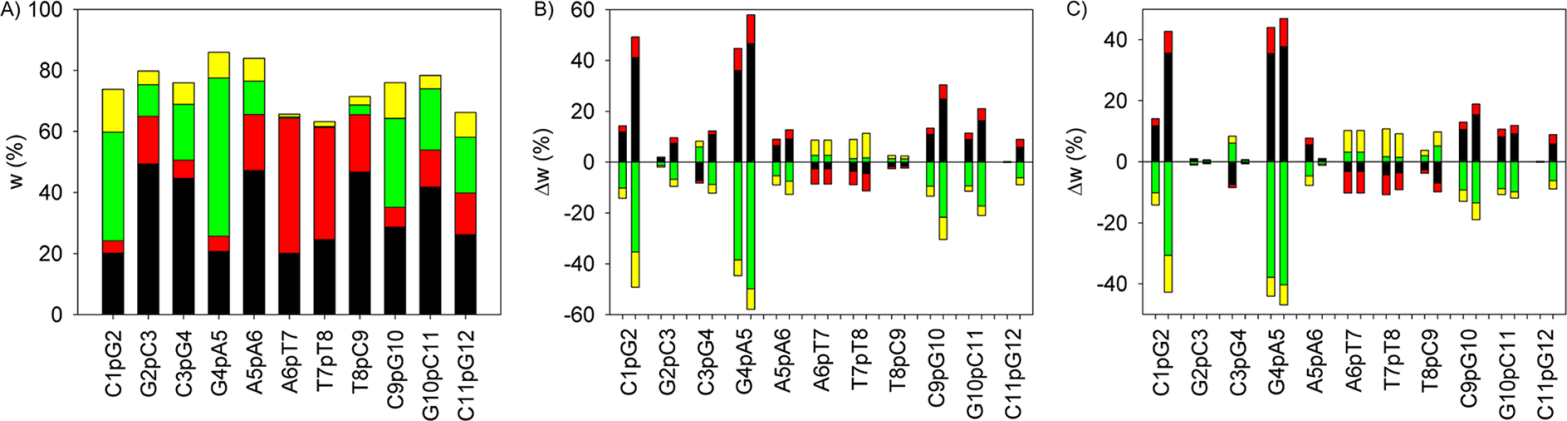
Population weights of BB00 (black), BB01 (red), BB04 (yellow), and BB07 (green) NtC phosphates in DNA. (**A**) Original *w* weights with the OL21 force field. (**B**) Differential Δ*w* weights due to fit to ^3^ *J*_P,H3’_ (left histograms) and δ_31P_ (right histograms) experimental data. (**C**) Differential Δ*w* weights fitted, including phosphate solvation/Mg^2+^. Δ*w* = *w* (OL21) – *w* (fitted).

The BI′ and BII′ populations were first fitted to experimental ^3^ *J*_P,H3’_ coupling constants (Table S12), which also adjusted the A6pT7 reference for δ_31P_ shifts. Subsequently, populations were refined using experimental δ_31P_ data (Table S13), assuming the calculated spectral parameters equal the experimental values (see eqs 3–5, Methods).

The MAD for ^31^P shifts decreased to 0.50 ppm after NtC population fitting, and further to 0.22 ppm when solvation effects were included (Table 3). Similarly, the MAD for ^3^ *J*_P,H3’_ couplings was reduced to 1.16 and 0.77 Hz, respectively (Table 4).

Population fitting led to slightly increased BII′ populations in the DNA stem region (6p7p8) and markedly decreased BII′ populations in tail phosphates (1p2, 4p5, 9p10p11) (Figure 4B). The BII′ population reduction was mitigated when solvation was incorporated (Figure 4C). The need for refinement of other NtC states was particularly evident in the 1p2 phosphate (Figure 3A,B), which showed irregular dynamics involving massively other NtC states outside the BI’/BII’ pool. Similar irregular NtC states were also captured in termini-adjacent phosphates 3p4, 9p10, and 11p12. Notably, population fitting by experimental data considerably improved spectral parameters, which indicated that refining canonical BI (BB00) and BII (BB07) states together with the BI–BII intermediate (BB04) is beneficial for most of the DNA phosphates (Figure 5).

**Figure 5:**
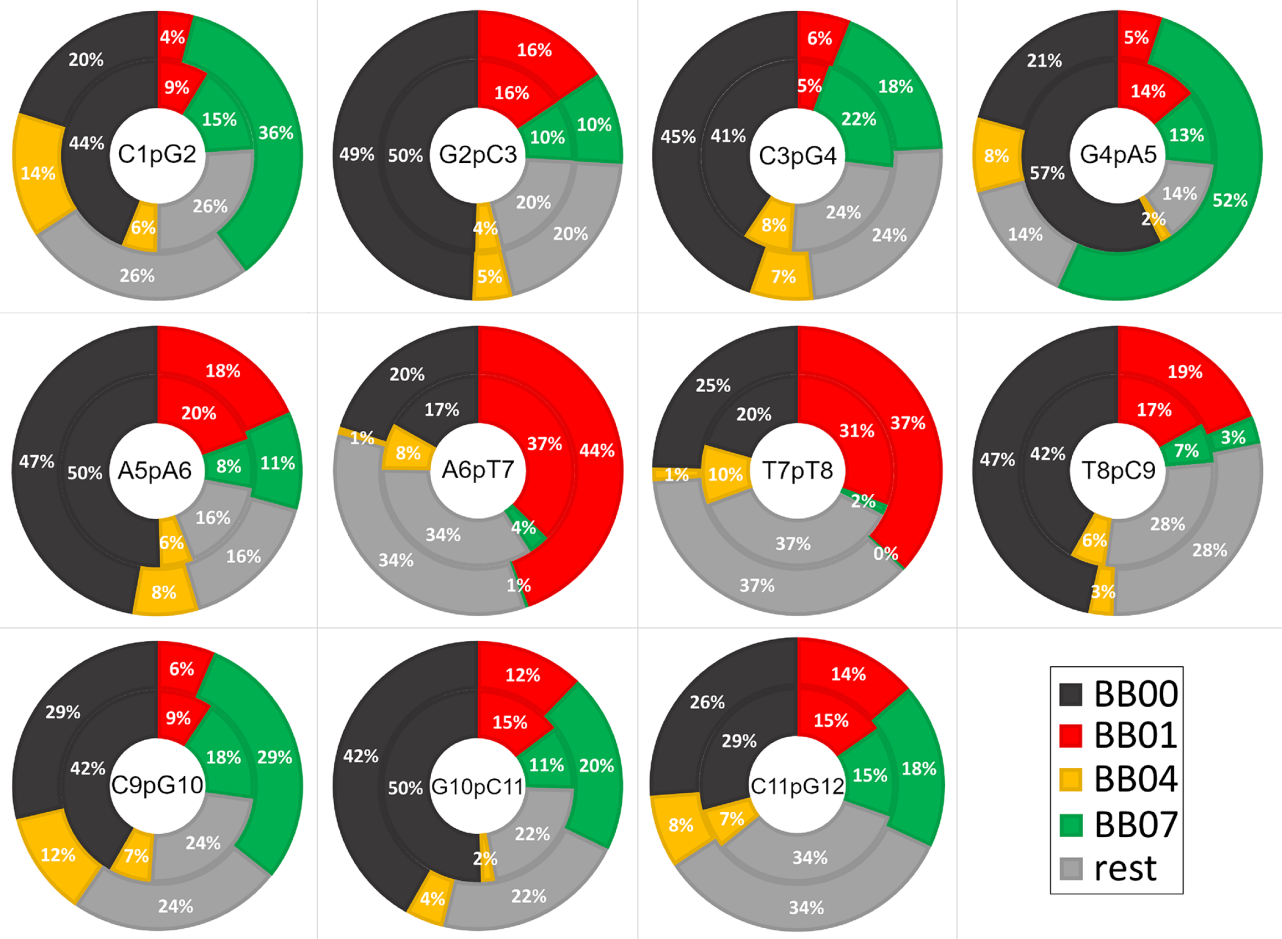
Populations of NtC phosphates calculated with the OL21 force field (the outer ring) and fitted to experimental data (the inner ring). Average NtC populations, as fitted with δ_31P_ and ^3^ *J*_P,H3’_ experimental data sets. The remaining NtC population (rest) was calculated with the OL21 force field.

### DNA Equilibrium Sustainability due to Temperature

Temperature-induced changes in the ^31^P shifts were generally negative (Figure 2C). An exception was measured for the 4p5 phosphate, whose ^31^P shift increased from −2.71 ppm (10 °C) to −2.68 ppm (30 °C) before decreasing to −2.70 ppm at 35 °C. Larger temperature variations (≥0.10 ppm) in the 1p2 (0.12 ppm), 3p4 (0.17 ppm), 7p8 (0.14 ppm), and 11p12 (0.14 ppm) phosphates (Table S4) suggested substantial temperature-dependent changes in DNA backbone dynamics.

Calculated ^31^P shifts (Figure S16, Tables S14, and S15) using both population-weighting and probability-averaging methods showed dependence on NtC population redistributions with temperature (Figure 6). The largest discrepancy between the two averaging schemes (0.26 ppm) was found in the 1p2 phosphate (Table S16), reinforcing its prominent conformational flexibility upon temperature.

**Figure 6:**
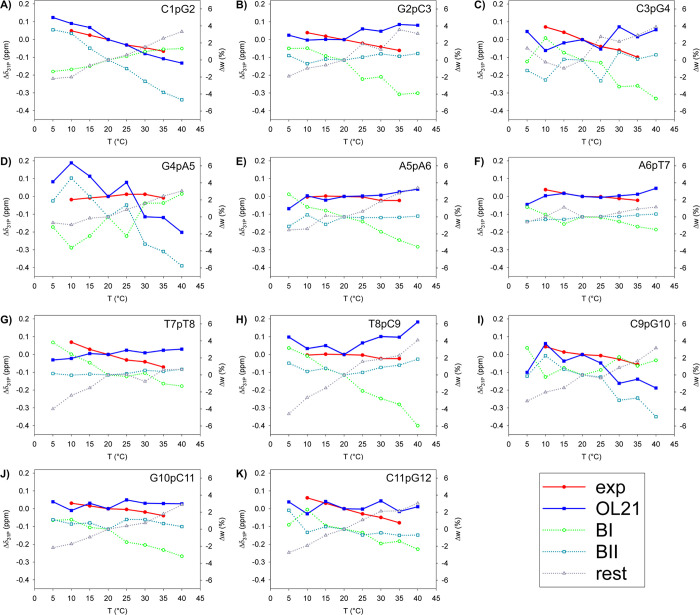
Dependences of δ_31P_ shifts and *w* population weights on temperature relative to parameter values at 20 °C calculated for C1pG2 (A), G2pC3 (B), C3pG4 (C), G4pA5 (D), A5pA6 (E), A6pT7 (F), T7pT8 (G), T8pC9 (H), C9pG10 (I), G10pC11 (J), and C11pG12 (K) DNA phosphates. The relative chemical shifts Δδ_31P_ measured (exp) and population-weighted averaged with the OL21 force field (OL21). The sectional probability weights for BB00 and BB01 (BI), BB04 and BB07 (BII) and remaining (rest) NtC states. Δδ_31P_ = δ_31P_ (*T*) – δ_31P_ (20 °C), Δ*w* = *w* (*T*) – *w* (20 °C).

Downfield shifts with increasing temperature in the 1p2, 5p6p7, and 9p10p11p12 phosphates qualitatively matched the experimental data. Scattered population redistributions indicated instability of particular NtC states in DNA phosphates: BI–BII (Figure 6D), BI–residual (Figure 6B), and BI–BII–residual (Figure 6C,I). Largely scattered population redistributions in the 4p5 phosphate (Figure 6D) revealed local instability in force field descriptions, contrasting with smoother population trends in the neighboring phosphate 5p6 (Figure 6E). Notably, the 4p5 phosphate, bridging structurally dissimilar DNA regions, exhibited the largest calculated shift variation 0.44 ppm and the only upfield shift measured upon temperature. The largest measured shift variation of 0.17 ppm was in the adjacent 3p4 phosphate (Table S4). Similarly, scattered population redistributions were calculated in the 9p10 phosphate (Figure 6I), where spectral parameters also deviated from the experiment (Figure 3A,B). These findings suggest that erratic redistributions of NtC populations, particularly in critical junction phosphates (e.g., 4p5, 9p10), signal force field instability. Consistent and well-behaved NtC transitions with temperature could serve as a criterion for evaluating the force field performance.

^31^P shifts dependences with the bsc1 force field (Figure S18) in 2p3 and 8p9p10 phosphates agreed qualitatively better with experiment than dependences with the OL21 force field. However, neither OL21 nor bsc1 force fields described the temperature dependences of NtC populations systematically better. Deficient descriptions of the critical junction phosphates common to both force fields indicated fundamental similarities of both parametrizations.

## Discussion

The assignment of NMR parameters to experimental DNA structures highlighted systematic differences between the X-ray and NMR structural models in the PDB repository. In crystal structures, DNA tail phosphates typically adopted canonical BI or BII conformations, whereas stem regions were more uniformly BI-like. NMR structures, in contrast, predominantly featured BI-like conformers throughout, which alone could not account for the observed variations in ^31^P chemical shifts and ^3^ *J*_P,H3’_ couplings.

NtC classification provided a refined structural framework that improved the interpretation of spectral parameters and offered a more detailed view of DNA conformational polymorphism. However, spectral parameters derived from different crystal structures showed greater variability than experimental NMR values, suggesting that individual crystal snapshots may not fully represent the dynamic equilibrium states of DNA. The equilibrium distribution likely comprises many thermodynamically allowed states, which, to a first approximation, might be described with an ensemble of prominent DNA structures captured in the crystal.

Whereas classical BI/BII categorization relies solely on the ε and ζ torsion angle orientations, the NtC classification incorporates all backbone torsions. This, in principle, better resolved conformational landscape enabled spectral assignments beyond the traditional bimodal model (Figure S19), improving particularly the resolution of structural intermediates and junctions between canonical conformational domains.

Our interpretation of NMR data incorporated both NtC population distributions and torsional dynamics using the population-weighted and probability-averaged methods. Dynamically averaged parameters showed significantly improved agreement with experiment compared to static models resolved by X-ray and NMR, and this agreement improved further when explicit solvation/Mg^2+^ was considered. Among the tested force fields, OL21 demonstrated better agreement with experimental data than bsc1, although neither provided the NtC populations in full agreement with experiment.

By fitting NtC populations to experimental data, we achieved a refined model of the Dickerson–Drew DNA equilibrium. This refined equilibrium featured reduced BII-like NtC populations in prominent tail phosphates, such as 4p5 and 9p10 phosphates, particularly in bridging regions that connect the structurally dissimilar DNA stem with the more flexible DNA terminal regions.

The temperature dependence of ^31^P chemical shifts provided a highly sensitive probe of local DNA dynamics and force field performance. Notably, phosphates at the junctions between dissimilar DNA domains (e.g., 4p5 and 9p10) displayed scattered and erratic changes in NtC populations, indicating force field instabilities. The 4p5 phosphate exhibited the only measured increase in ^31^P chemical shift with rising temperature, an anomaly that reflected both its pivotal structural role as a junction and its dynamic lability.

These observations suggest that the temperature dependence of NtC populations in structurally transitional regions could serve as a stringent benchmark for future force field upgrades. Ensuring consistent and physically realistic redistribution of conformational substates under changing conditions due to temperature is essential for achieving reliable simulation-based interpretations of experimental data.

## Conclusions

The Dickerson–Drew DNA equilibrium was elucidated through a structure-dynamics-based interpretation of ^31^P NMR parameters. Unconstrained MD simulations with OL21 and bsc1 force fields, aided by NtC classification, enabled reliable dynamic averaging of spectral parameters beyond the conventional BI/BII framework.

Neither force field fully reproduced experimental data, and deviations of spectral parameters from experiment due to NtC population distributions indicated shortcomings in their ability to capture conformational equilibria at specific phosphate sites. Refining NtC populations based on experimental NMR data revealed discrepancies, particularly in the representation of BII-like states, especially in DNA tail phosphates bridging the BI-like stem and the DNA terminal regions.

Temperature-dependent analyses of ^31^P chemical shifts provided a sensitive metric for detecting local conformational transitions and highlighted deficiencies in current force fields in a one-to-one relation with NtC states populations. Critical junctional phosphates such as 4p5 and 9p10 exhibited erratic NtC population redistributions due to temperature, suggesting imbalanced force field performances due to improper balance of structure-dynamically dissimilar adjacent domains.

The integration of experimental NMR data with the NtC structural annotation represents a robust strategy for evaluating and refining DNA force fields. This methodology supports the development of next-generation force fields capable of faithfully reproducing the structural dynamics of DNA under physiologically relevant conditions.

## Supplementary Material



## Notes

Calculated spectral parameters for NtC phosphates and experimental PDB structures, geometrical dependences of spectral parameters, MD-calculated probability distributions of DNA backbone torsion angles, geometries of solvated NtC phosphates, spectral parameters contributions due to NtC populations, calculated differential spectral parameters for NMR-equivalent DNA phosphates, temperature dependences of δ_31P_ shifts, calculated δ_31P_ shifts for NtC phosphates categorized as BI or BII, spectral parameter values measured in different experiments, δ_31P_ shifts temperature dependences, calculated geometrical and spectral parameters and population weights for NtC phosphates, NtC classification and spectral parameters for experimental PDB structures, and calculated spectral parameters (PDF)

## Abbreviations

NMR: nuclear magnetic resonance
MD: molecular dynamics
