# Supplementary material for: Deciphering Dickerson–Drew DNA Equilibrium beyond the BI/BII DNA Dichotomy by Interpretation of 31P NMR Parameters
Source: J Chem Theory Comput. 2025 Sep 16;21(19):10006–17. doi: 10.1021/acs.jctc.5c01076 (PMC12529900; doi:10.1021/acs.jctc.5c01076)
Supplement: Supplementary file 1 [file ct5c01076_si_001.pdf]

MANUSCRIPT TITLE

Deciphering Dickerson-Drew DNA Equilibrium Beyond the BI/BII DNA Dichotomy by ^{31}P NMR Parameters Interpretation

AUTHORS
Jiří Fukal[1,2], Miloš Budešínský[1], Jakub Šebera[3], Marie Zgarbová[2], Petr Jurečka[2], Vladimír Sychrovský[1,*]

[1] Institute of Organic Chemistry and Biochemistry, Czech Academy of Sciences, 166 10 Praha 6, Czech Republic
[2] Department of Physical Chemistry, Faculty of Science, Palacký University Olomouc, 77146 Olomouc, Czech Republic
[3] J. Heyrovský Institute of Physical Chemistry, Czech Academy of Sciences, 182 00 Prague 8, Czech Republic

* To whom correspondence should be addressed. Email: sychrovsky@uochb.cas.cz

Supporting Information:

Supporting Figures S1 – S19: additional calculated NMR parameters S1 – S6, calculated dependences of NMR parameters on geometry S7 – S8, calculated probability distributions S9 – S10, calculated geometries of solvated phosphate S11, calculated dependences of NMR parameters on NtC classes S12 – S13, calculated differences of NMR-equivalent parameters in DNA S14 – S15, calculated temperature dependences of NMR parameters S16 – S18, 31P NMR shifts calculated in NtC classes S19.

Supporting Tables S1 – S16: experimental NMR parameters S1 – S5, geometrical and NMR parameters and populations of NtC classes S6, NtC classification of experimental DNA structures S6 – S8, NMR parameters assigned to experimental DNA structures S9 – S10, NMR parameters calculated in solvated NtC classes S11, NMR parameters and population weights fitted to experimental NMR data S12 – S13, calculated dependences of 31P NMR shifts on temperature S14 – S16.

Supporting References: supporting references 1-5.

Supplementary Figures S1 – S19

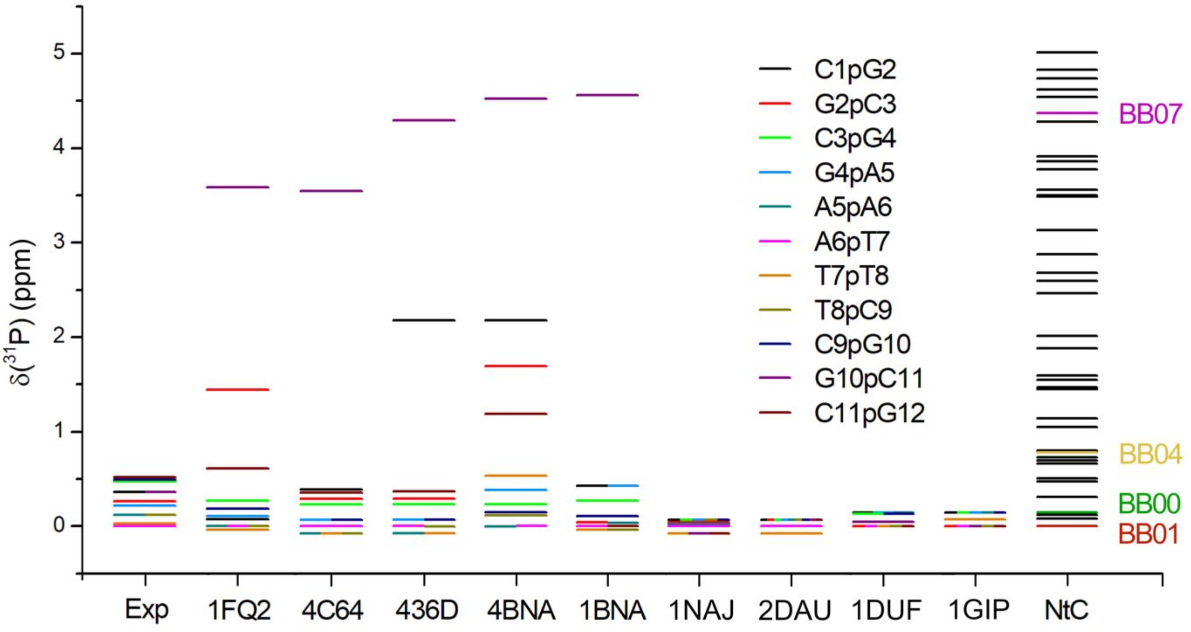

Figure S1. The δ_{31P} shifts measured (Exp), assigned in x-ray (PDB 1FQ2, 4C64, 436D, 4BNA and 1BNA) and NMR (PDB 1NAJ, 2DAU, 1DUF and 1GIP) structures and calculated in NtC phosphates (NtC). Average δ_{31P} shifts in NMR model structures (Figure S3) and DNA strands in X-ray structures (Figure S4). δ_{31P} shifts referenced to A6pT7 phosphate.

A)

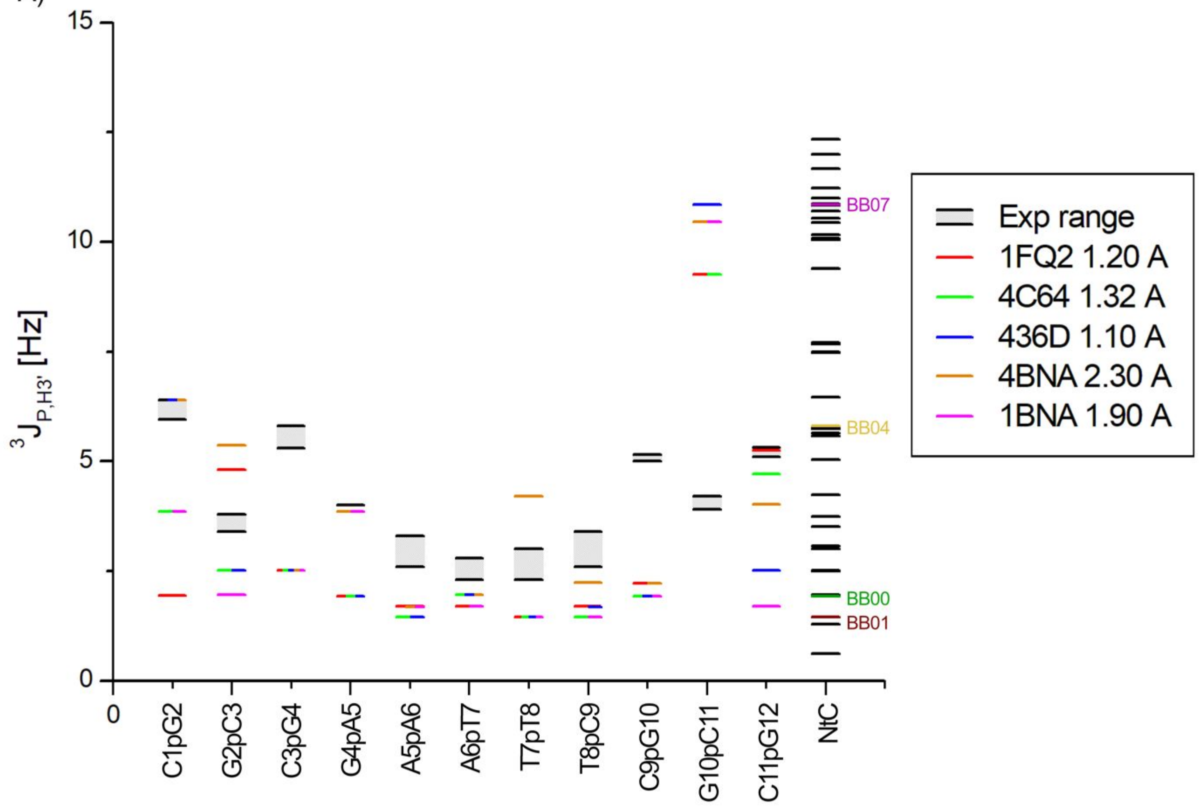

B)

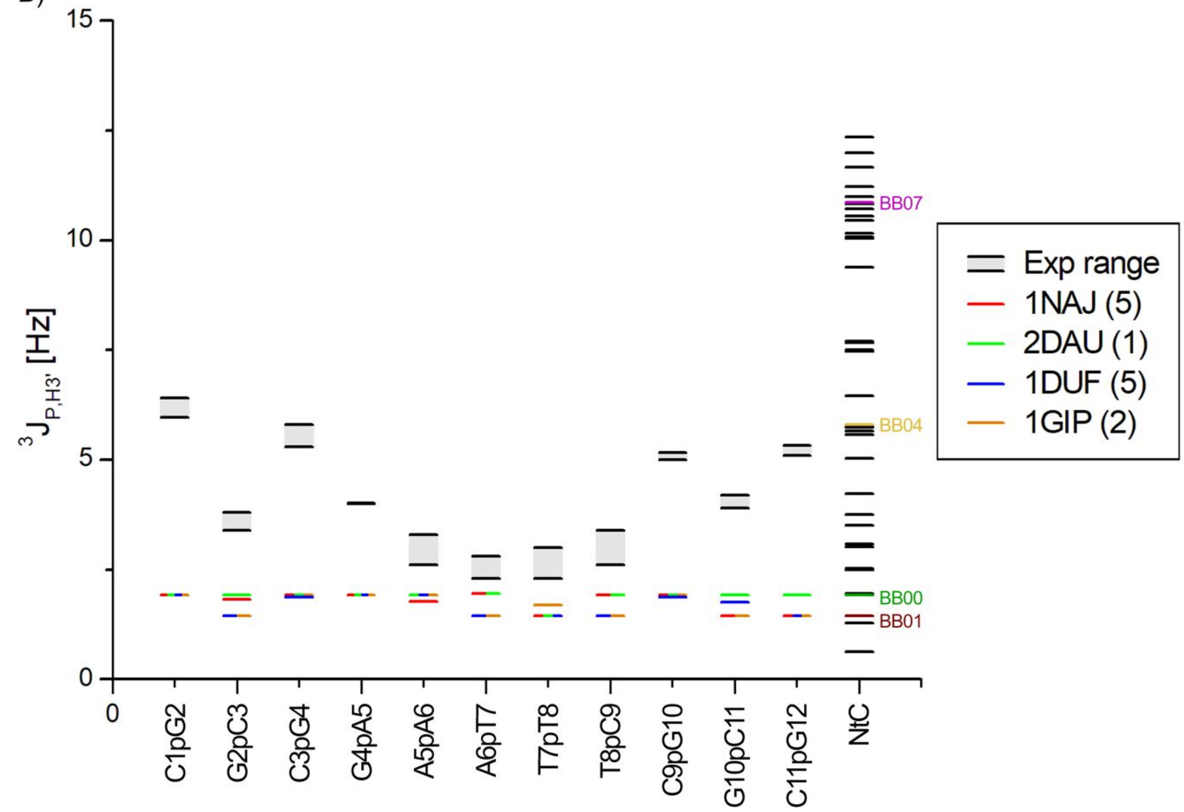

Figure S2. The $^3J_{PH3'}$ couplings measured (Exp range), assigned in x-ray (PDB 1FQ2, 4C64, 436D, 4BNA and 1BNA) and NMR (PDB 1NAJ, 2DAU, 1DUF and 1GIP) structures and calculated in NtC phosphates (NtC). Average $^3J_{PH3'}$ couplings of parameters assigned in NMR structural models and DNA strands in X-ray structures.

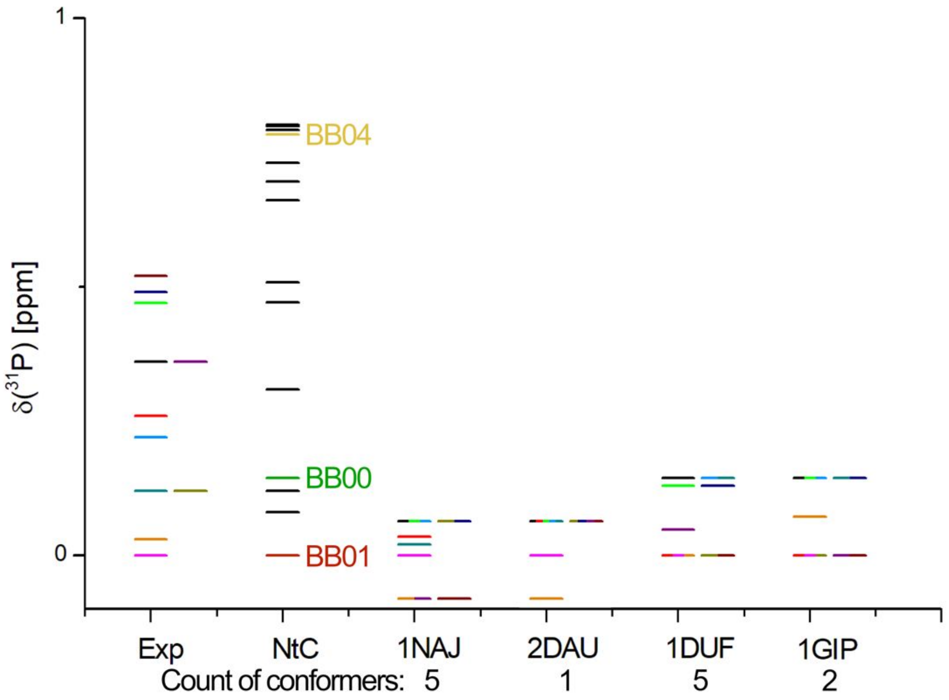

Figure S3. The δ_{31P} shifts measured (Exp), calculated in NtC phosphates (NtC) and assigned in NMR structures (PDB 1NAJ, 2DAU, 1DUF and 1GIP).

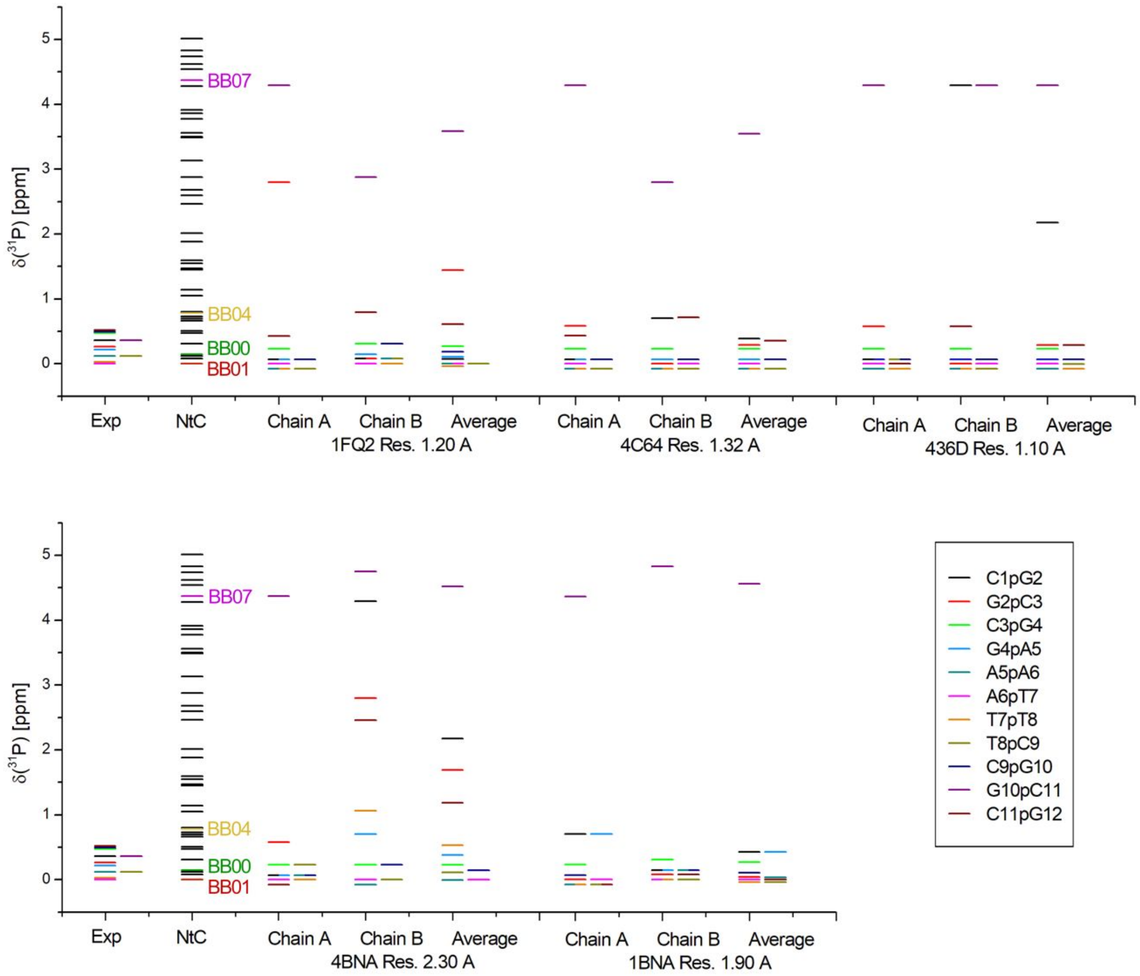

Figure S4. The δ_{31P} shifts measured (Exp), calculated in NtC phosphates (NtC) and assigned in A and B DNA strands in x-ray structures (PDB (1FQ2, 4C64, 436D, 4BNA and 1BNA).

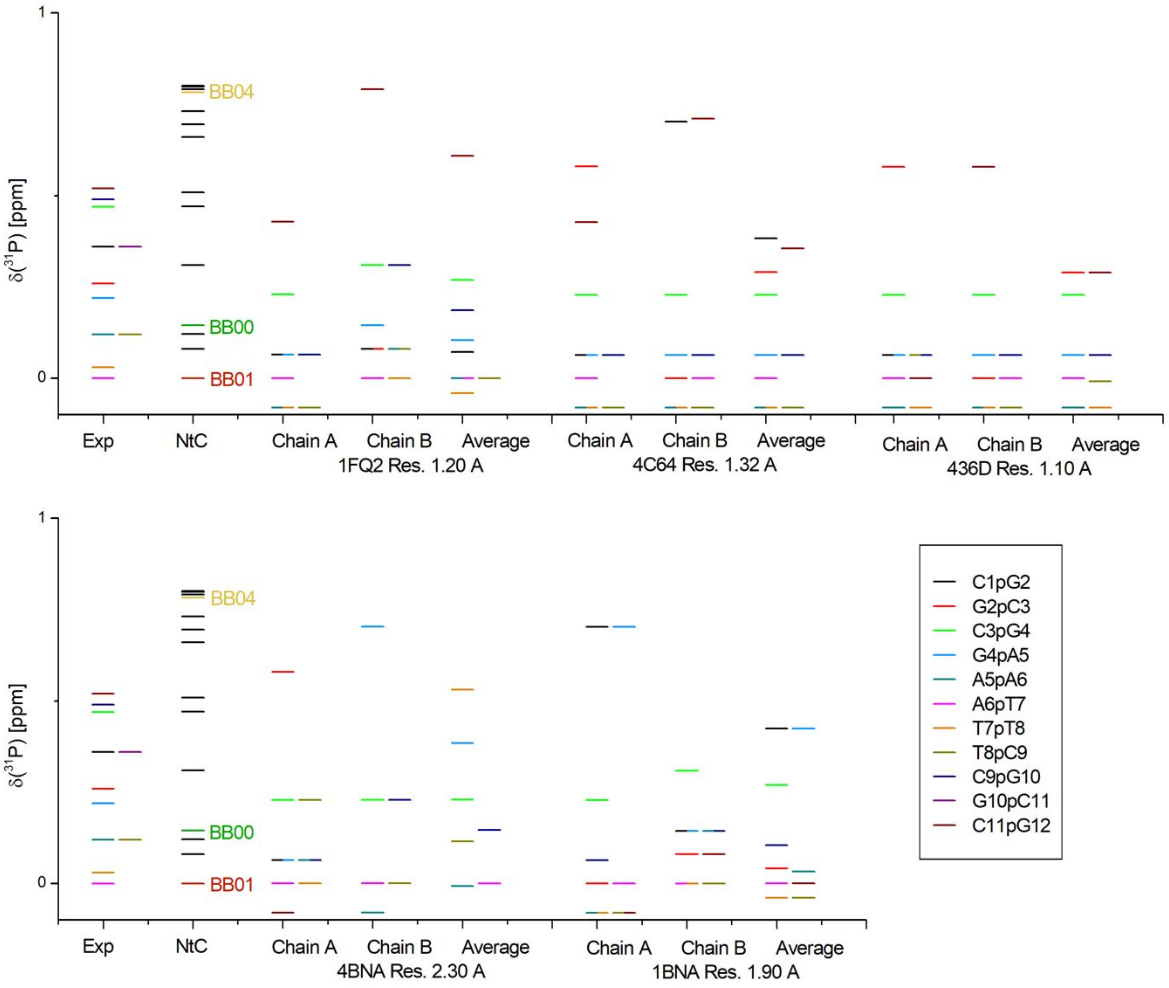

Figure S5. The δ_{31P} shifts (ppm): experiment (Exp), calculation in NtC classes (NtC) and calculation in A and B DNA strands in x-ray structures (PDB ID 1FQ2, 4C64, 436D, 4BNA and 1BNA).

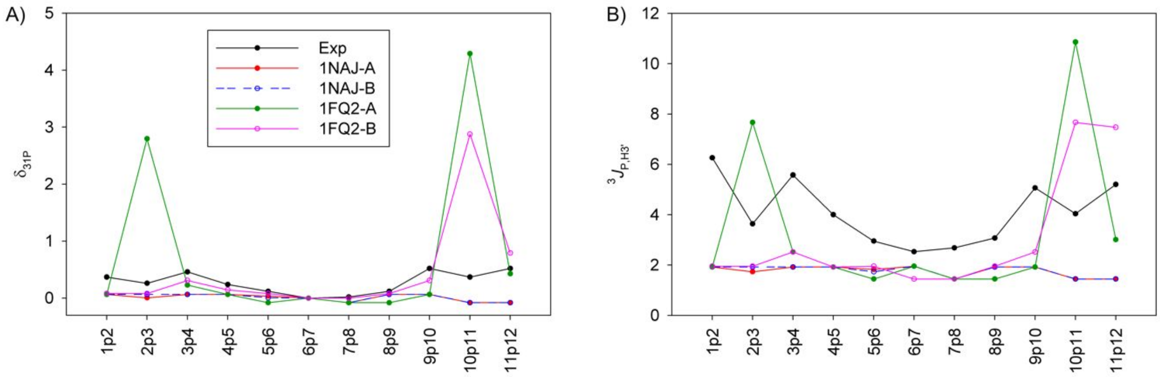

Figure S6. Representative comparison of the spectral parameters assigned in NMR and X-ray structures. The δ_{31P} shifts (ppm) (A) and $^{3}J_{P,H3'}$ couplings (Hz) (B) measured (Exp) and assigned in A and B DNA strands of 1NAJ NMR and 1FQ2 X-ray structures. The δ_{31P} shifts referenced to A6pT7 phosphate.

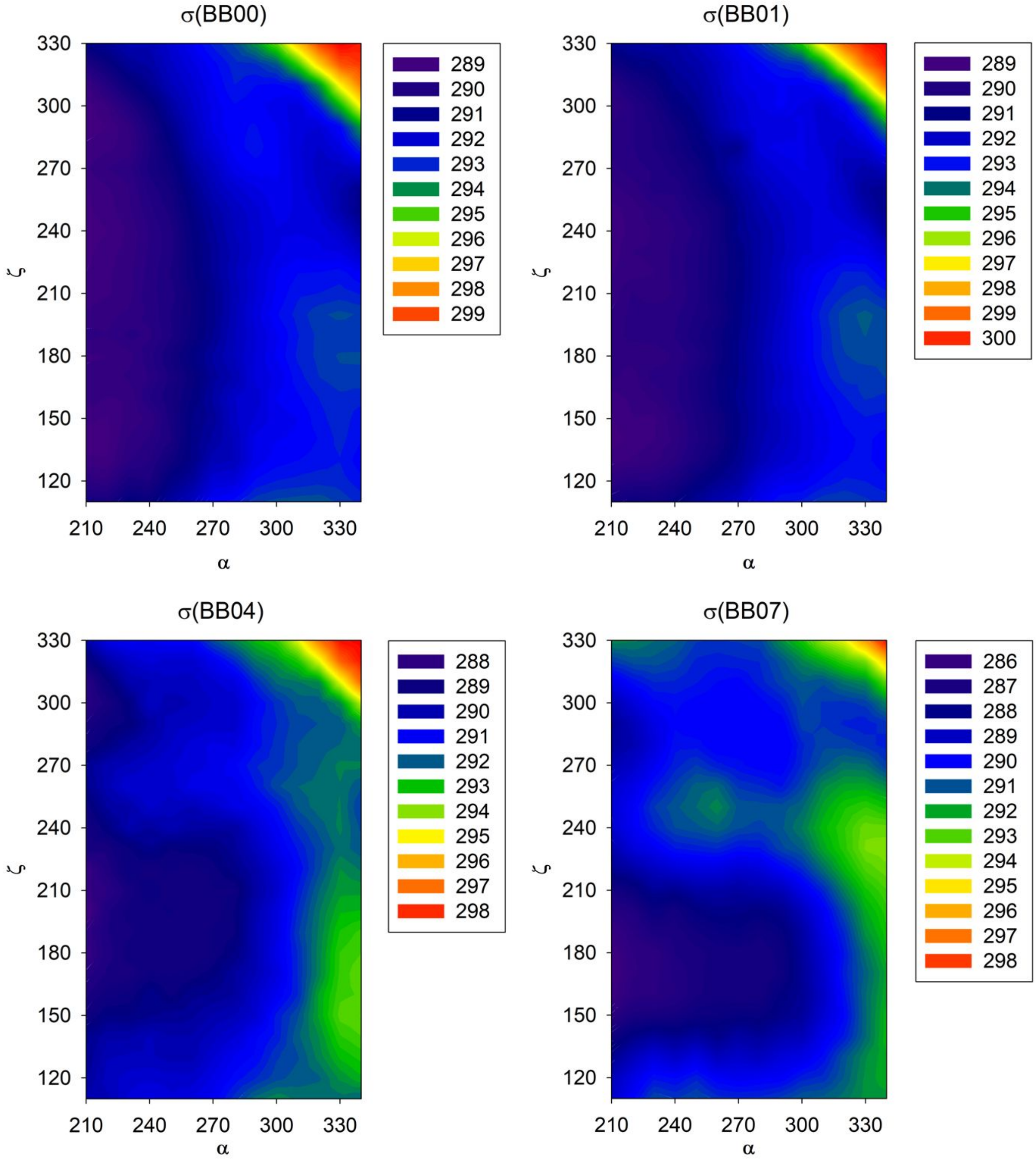

Figure S7A. The dependence of σ_{31P} shielding (ppm) on α and ζ torsion angles (°) calculated in BB00, BB01, BB04 and BB07 NtC phosphates.

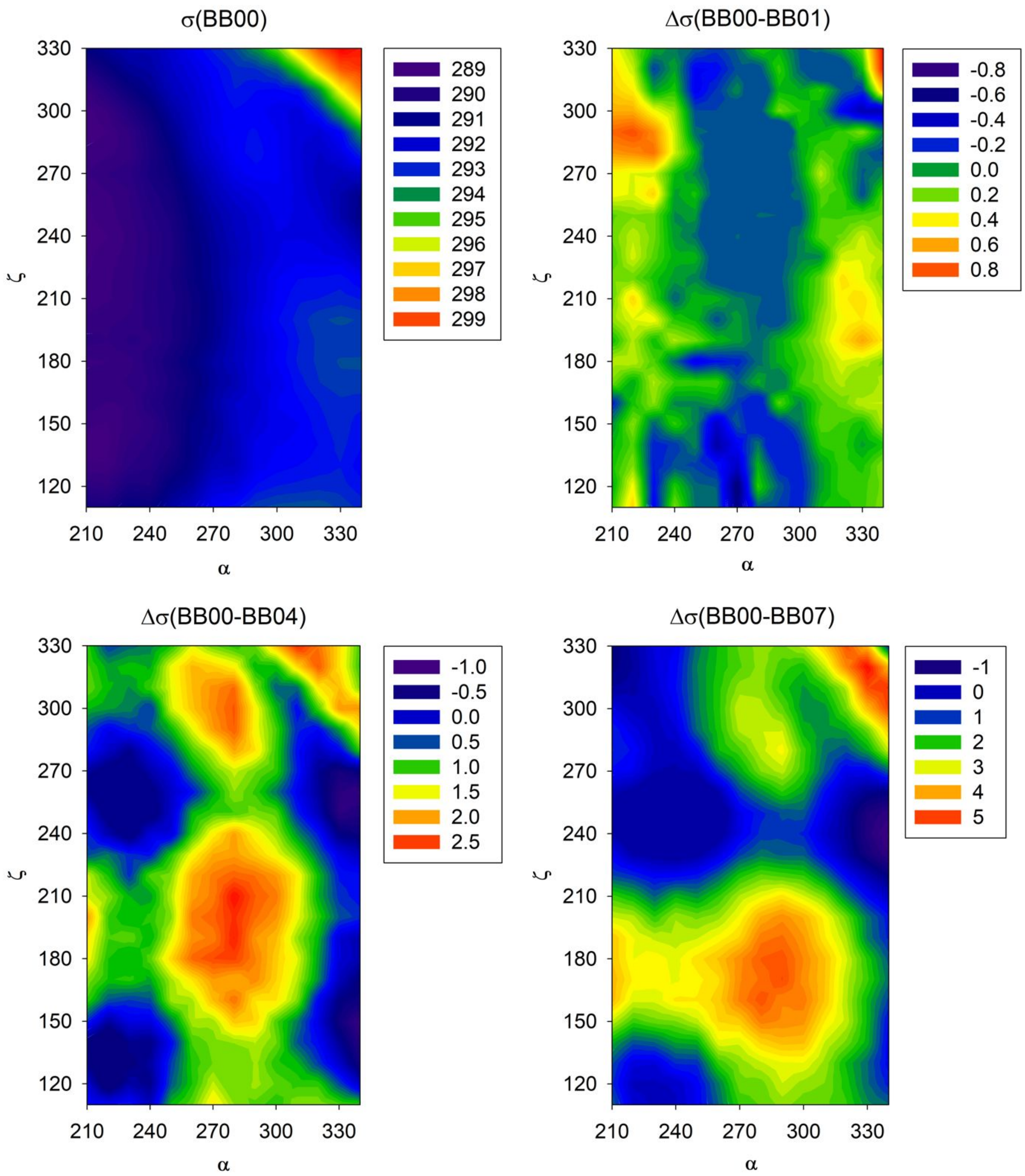

Figure S7B. The dependence of σ_{31P} shielding (ppm) on α and ζ torsion angles (°) in BB00 phosphate and dependences of δ_{31P} shifts relative to BB00 in the BB01, BB04 and BB07 phosphates.

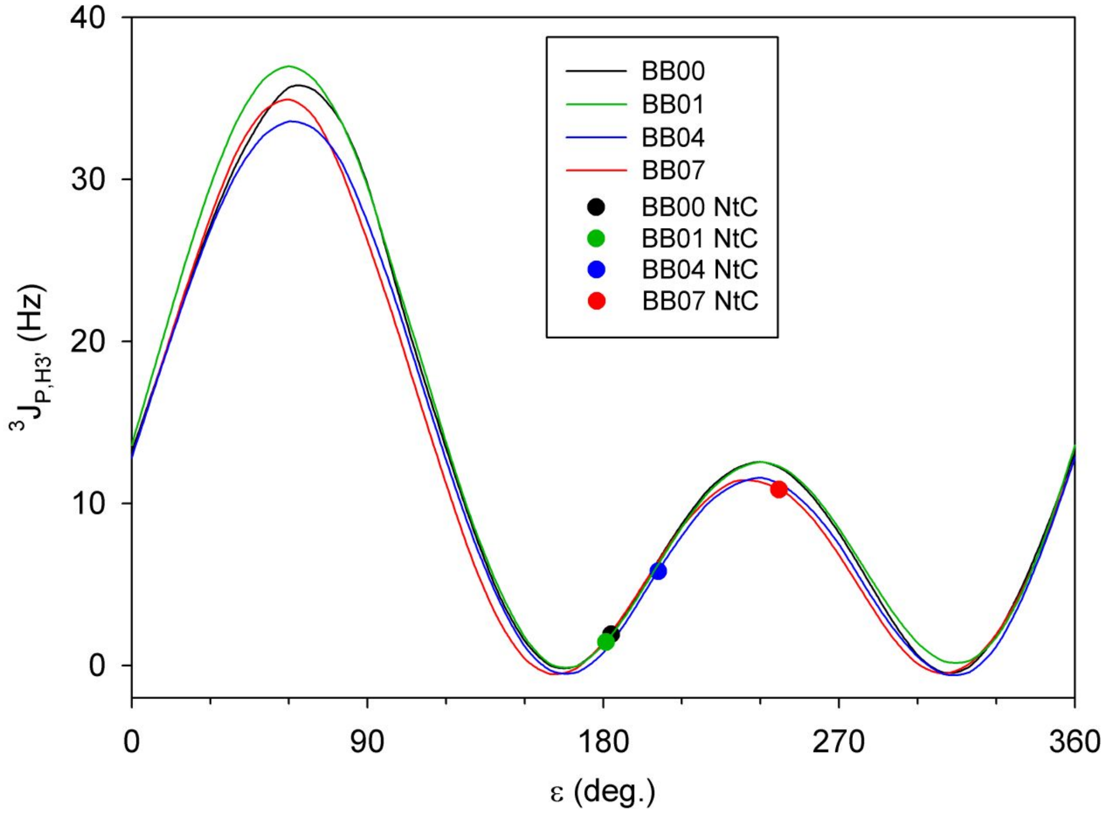

Figure S8. The dependence of $^3J_{PH3'}$ coupling on ε torsion angle calculated in BB00, BB01, BB04 and BB07 NtC phosphates and the $^3J_{PH3'}$ couplings in relevant NtC classes.

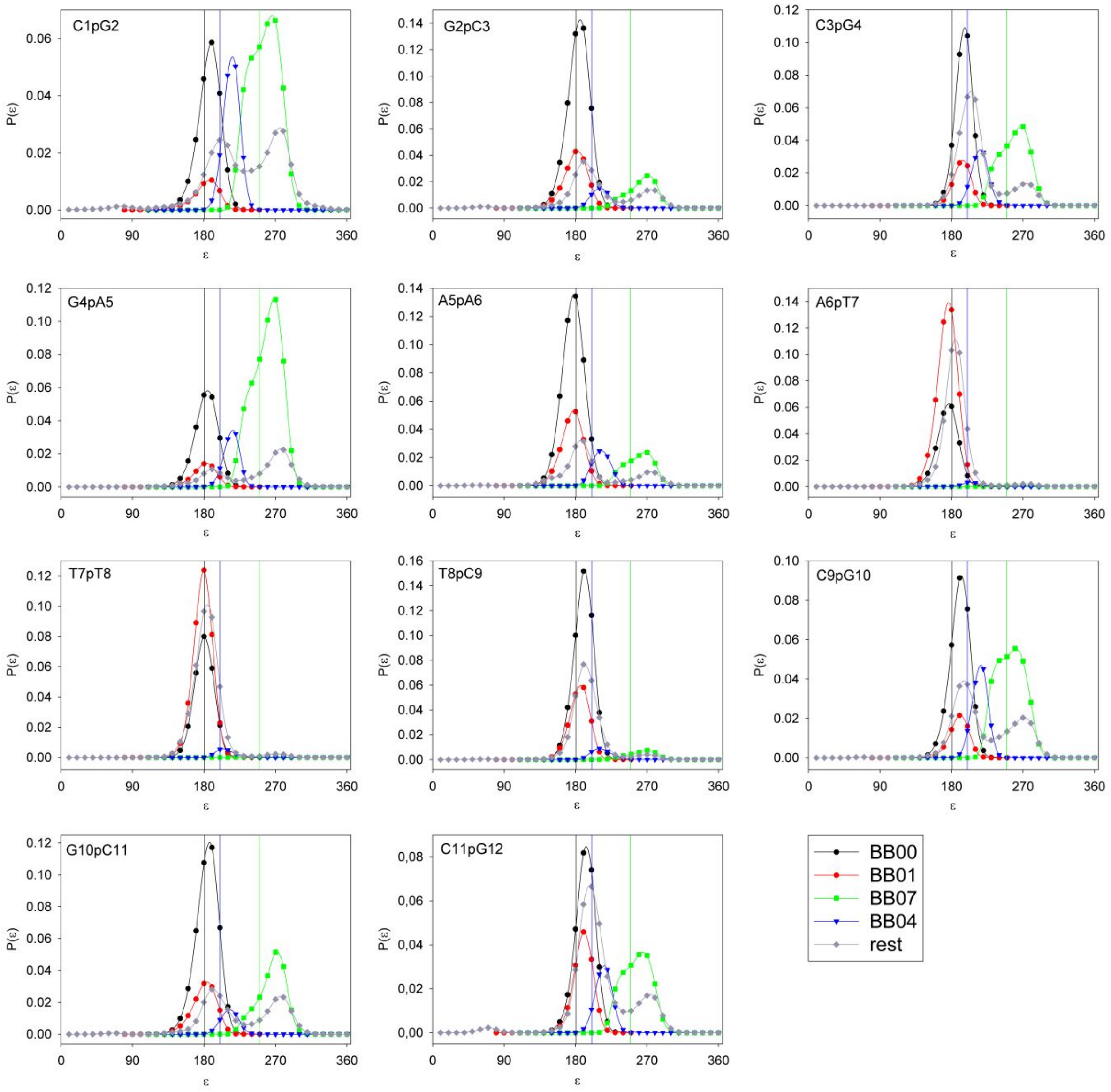

Figure S9. The sectional *P*(ε) probability distributions of ε torsion angle (deg.) in DNA phosphates calculated for BB00, BB01, BB04 and BB07 NtC states with the OL21 MD force field.

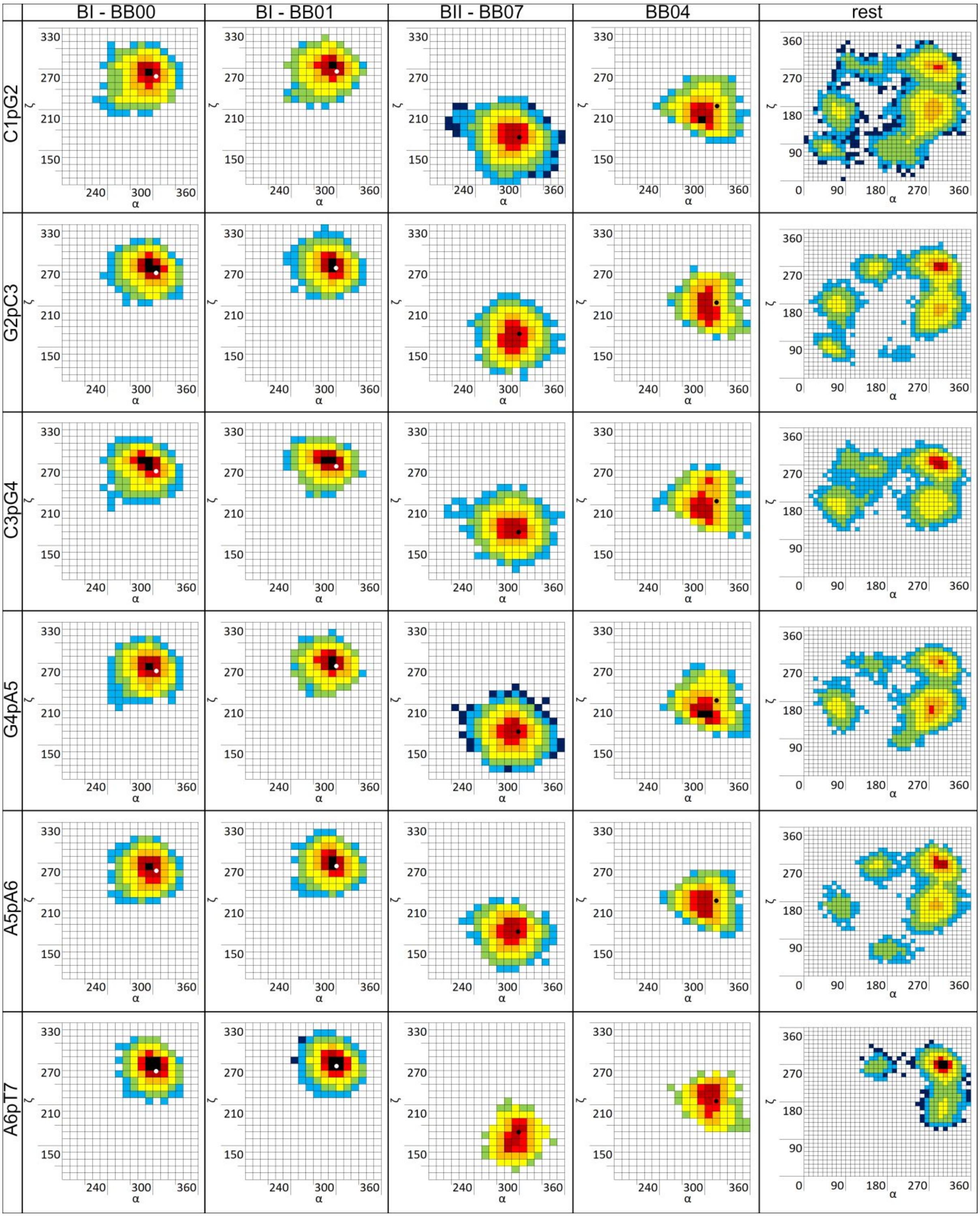

Figure S10A. The sectional $P(\zeta,\alpha)$ probability distributions of ζ and α torsion angles (deg.) in DNA phosphates calculated for BB00, BB01, BB04 and BB07 NtC states with the OL21 MD force field.

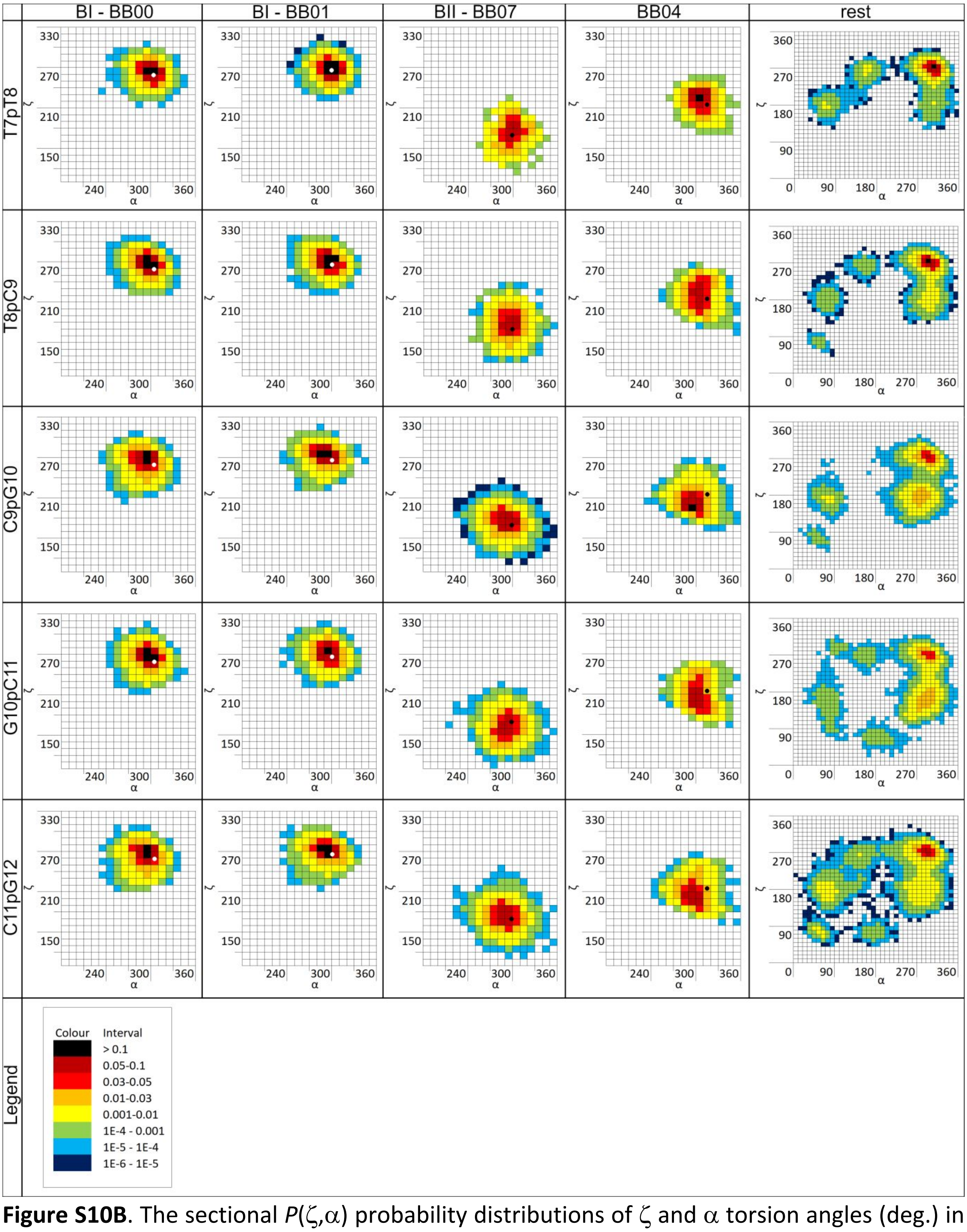

Figure S10B. The sectional *P*(ζ,α) probability distributions of ζ and α torsion angles (deg.) in DNA phosphates calculated for BB00, BB01, BB04 and BB07 NtC states with the OL21 MD force field.

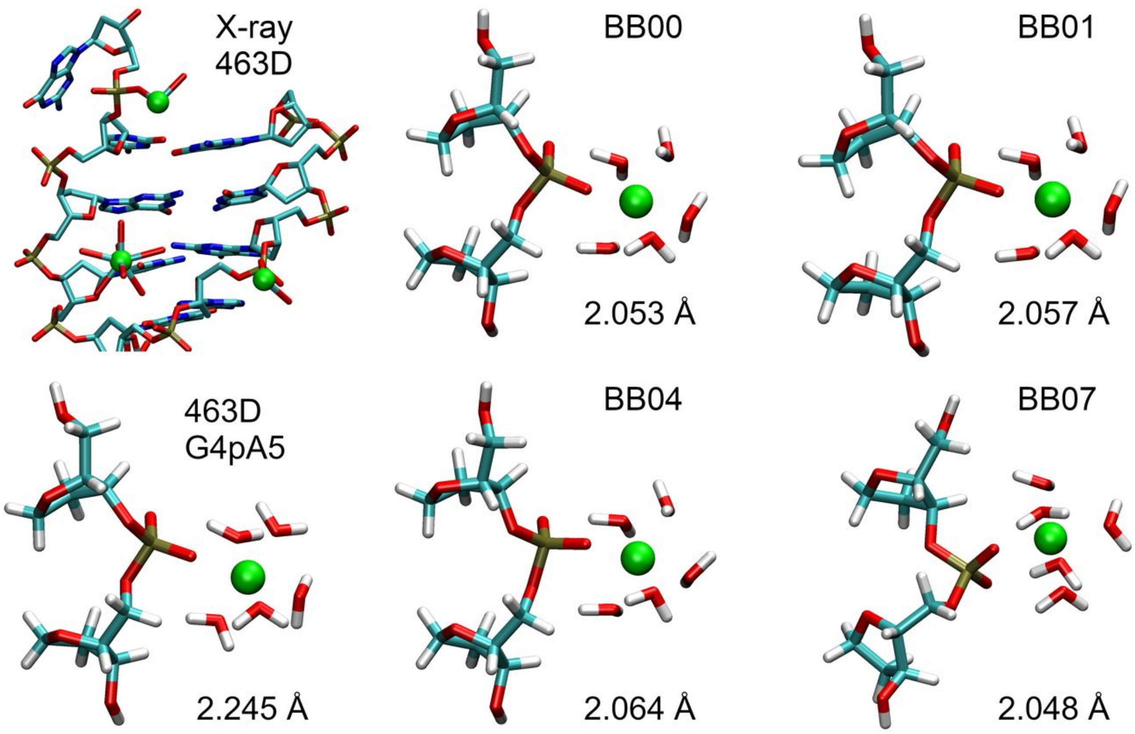

Figure S11. Solvated DNA phosphates. Preparation of structural models of solvated NtC phosphates: Ca^{2+} cation coordinated to G4pA5 phosphate in the 463D crystal structure was replaced by Mg^{2+}, four water molecules were added to one water molecule captured in the crystal to maintain six metal ligands, backbone torsion angles in the sugar-phosphate-sugar DNA fragments were oriented in compliance with their orientations in the four NtC classes and kept during geometry optimization (Methods, the main text), the Ahlrichs VTZ atomic basis was employed for Mg atom. Distances between the metal (green sphere) and nearest exo-oxygen atom of phosphate are in Å.

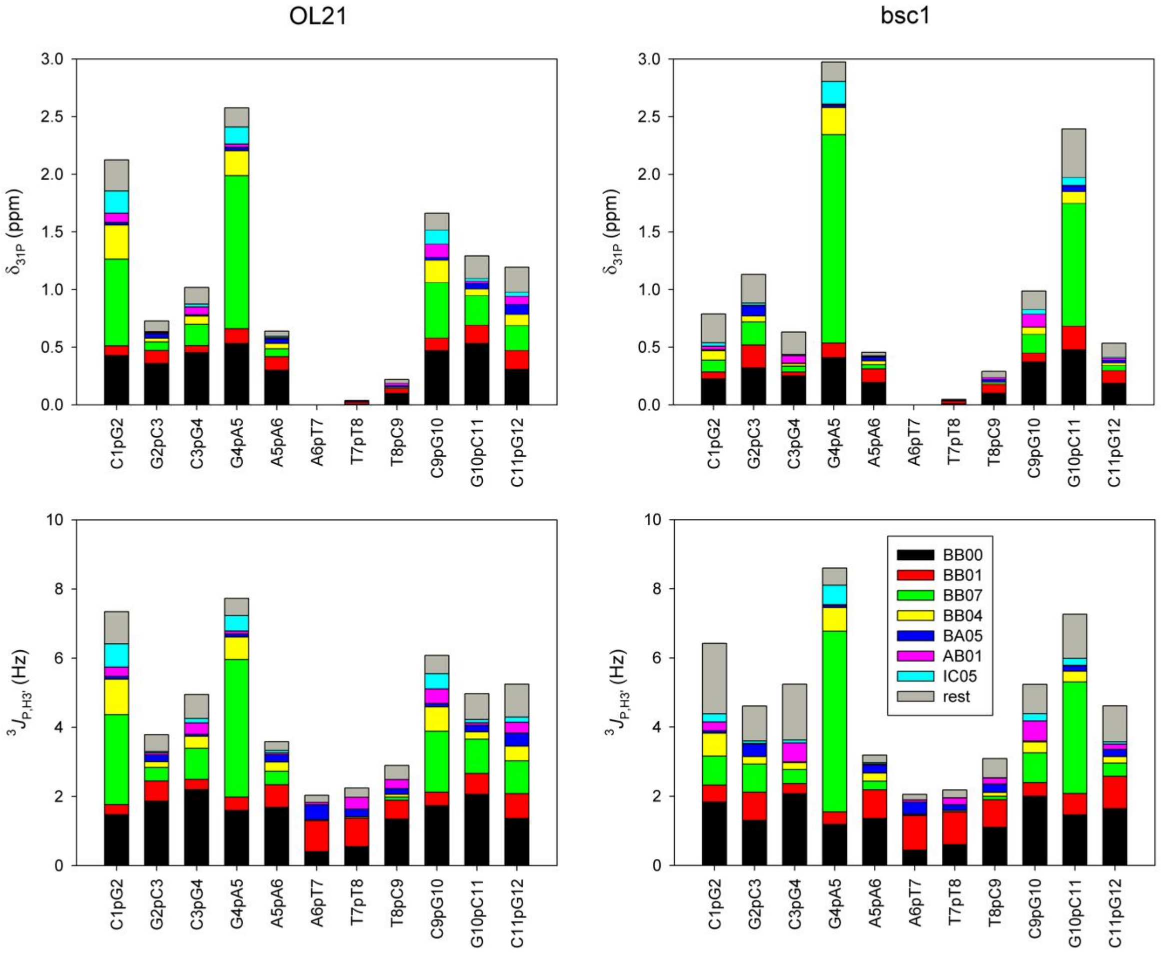

Figure S12. The δ_{31P} shifts and $^3J_{PH3'}$ couplings contributions due to NtC population weights calculated by Population weighting with the OL21 and bsc1 force fields.

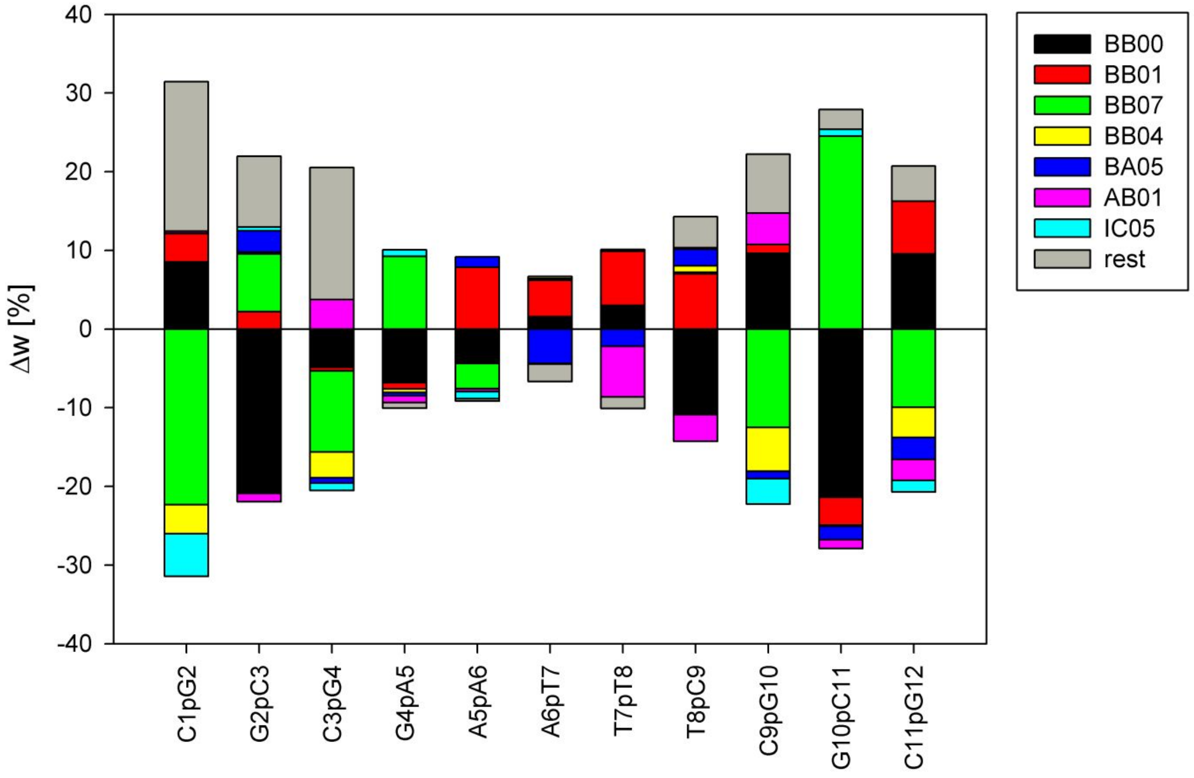

Figure S13. The differential NtC population weights with OL21 and bsc1 force fields in DNA. Differential population weight (Δw) was calculated as bsc1 weight minus OL21 weight. Residual weight (rest) was sum of weights of other NtC states.

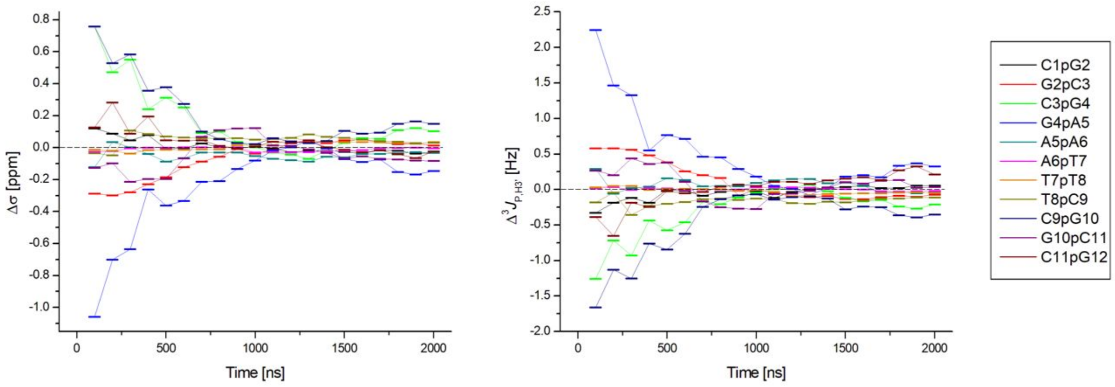

Figure S14. Cumulative differences of spectral parameters in NMR-equivalent phosphates calculated by population weighting with the OL21 force field.

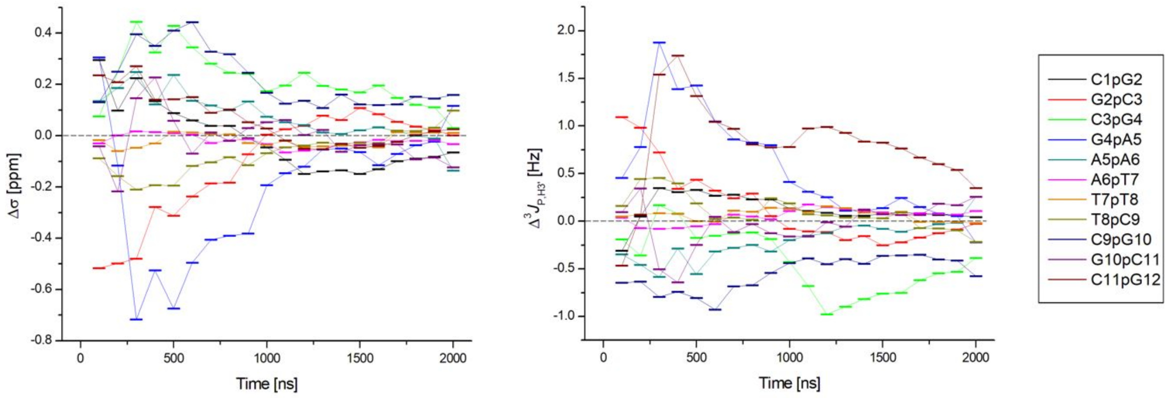

Figure S15. Cumulative differences of spectral parameters in NMR-equivalent phosphates calculated by population weighting with the bsc1 force field.

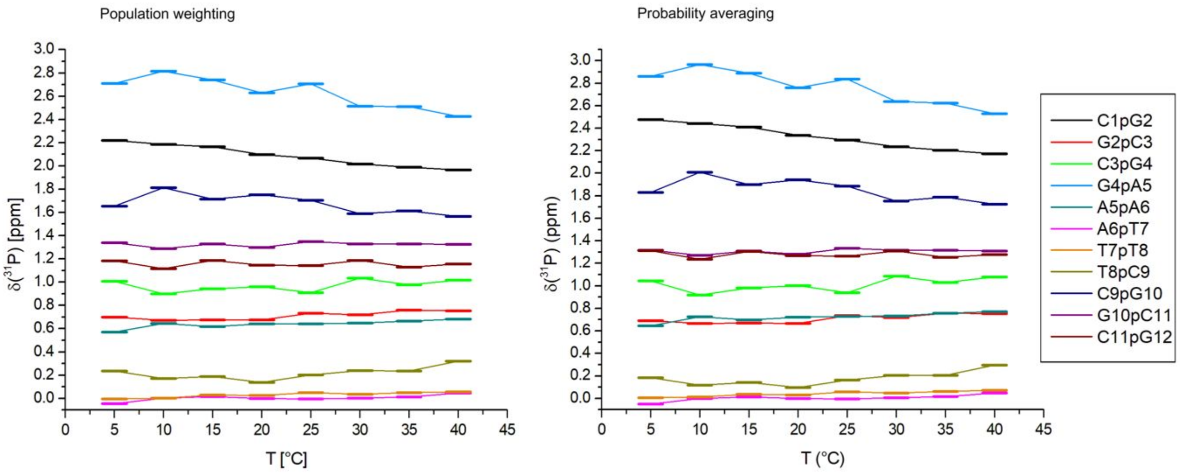

Figure S16. The dependences of δ_{31P} shifts calculated by the methods of Population weighting and Probability averaging on temperature.

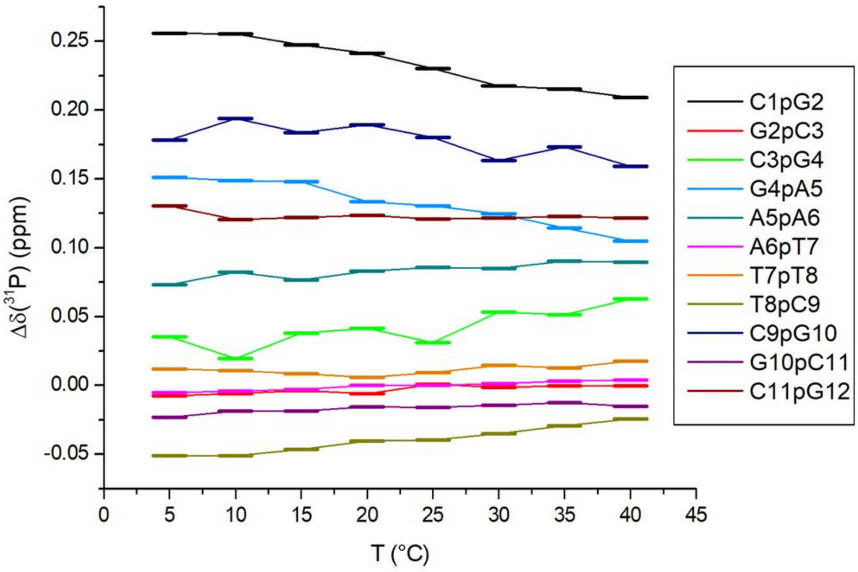

Figure S17. The solely effect on δ_{31P} shifts due to Probability averaging at different temperatures calculated as difference of δ_{31P} shifts by Population weighting minus δ_{31P} shifts by Probability averaging.

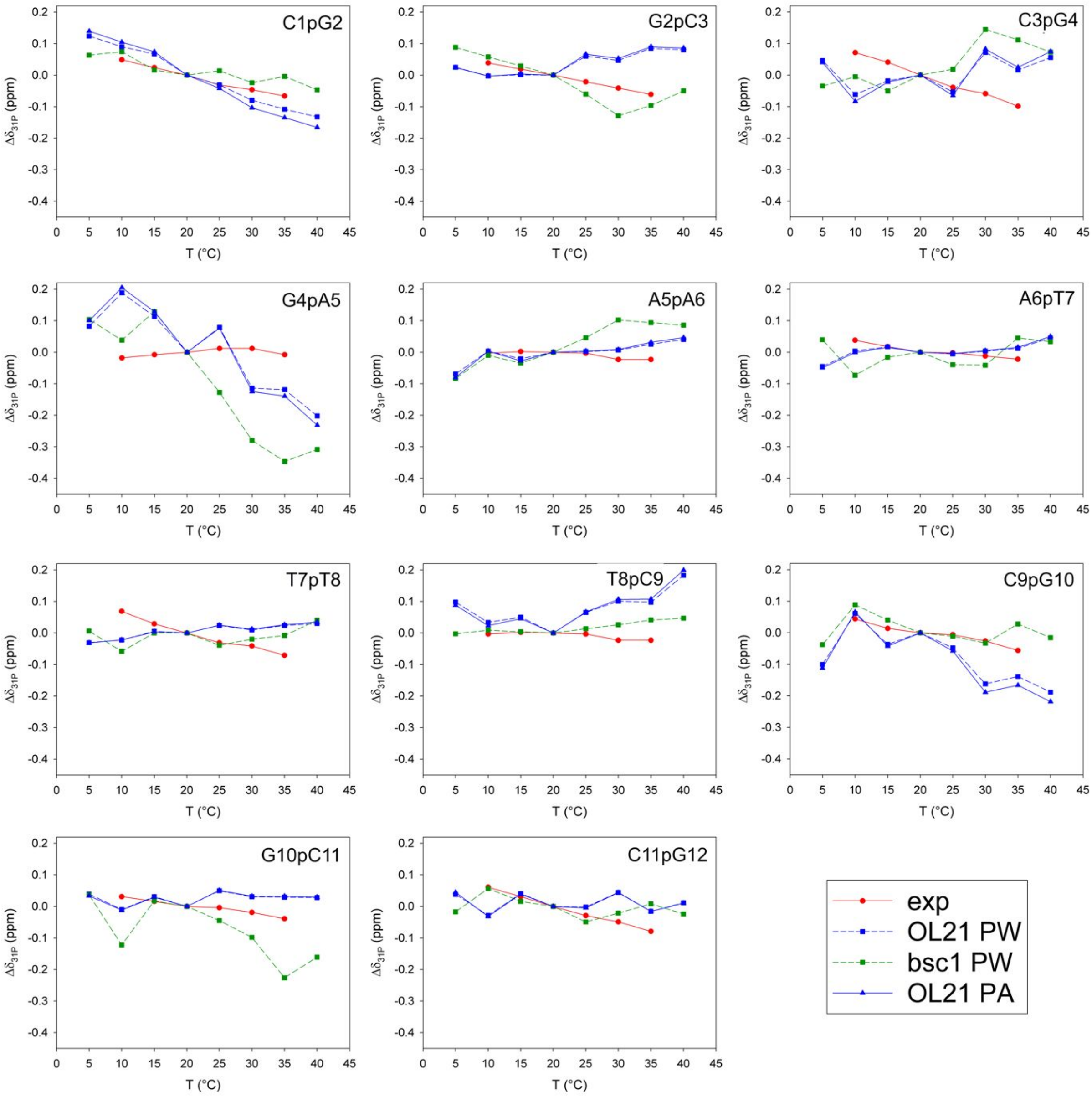

Figure S18. The dependences of δ_{31P} shifts on temperature measured (exp) and calculated by Population weighting (PW) and Probability averaging (PA) with OL21 and bsc1 force fields. δ_{31P} shifts related to δ_{31P} at 20 °C; $\Delta\delta_{31P} = \delta_{31P}$ (T) – δ_{31P} (20 °C).

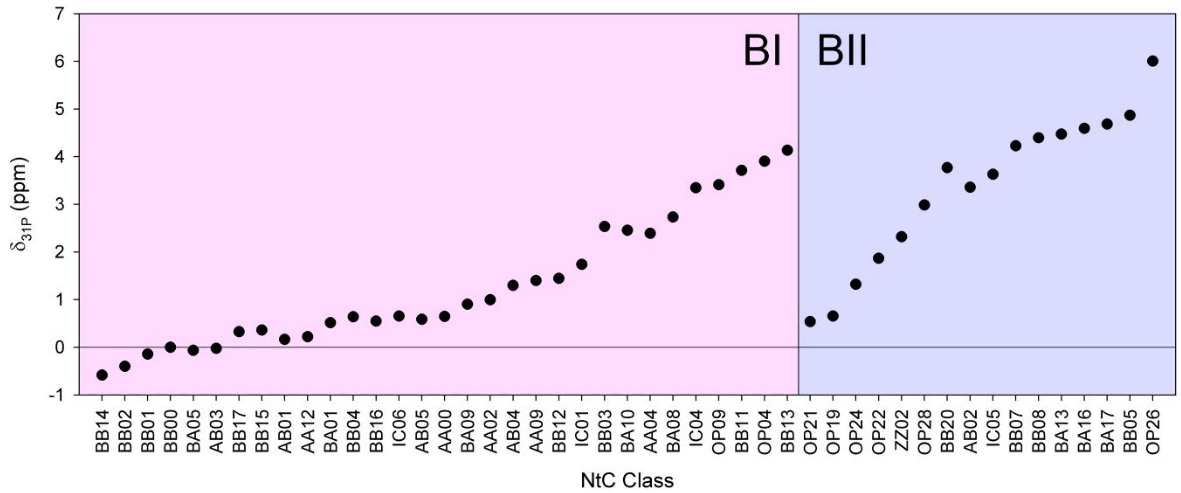

Figure S19. The δ_{31P} shifts calculated in NtC phosphates (Table S6) classified as BI or BII according to $\varepsilon - \zeta < 0$ (BI), $\varepsilon - \zeta \geq 0$ (BII).

Supplementary Tables S1 – S16

Table S1. The δ_{31P} shifts (ppm) measured in DNA.

DNA step	a)	b)	c)	d)	e)	f)
C1pG2	–2.52	–4.18	–4.14	4.94	4.93	–1.00
G2pC3	–2.64	–4.28	–4.26	4.81	4.79	–1.12
C3pG4	–2.42	–4.07	–4.09	4.97	4.95	–0.95
G4pA5	–2.69	–4.32	–4.24	4.87	4.85	–1.06
A5pA6	–2.80	–4.42	–4.35	4.70	4.69	–1.20
A6pT7	–2.92	–4.54	–4.49	4.57	4.59	–1.33
T7pT8	–2.89	–4.51	–4.48	4.59	4.62	–1.33
T8pC9	–2.80	–4.42	–4.37	4.72	4.76	–1.20
C9pG10	–2.39	–4.05	–4.02	5.09	5.13	–0.85
G10pC11	–2.56	–4.18	–4.15	4.93	4.97	–1.00
C11pG12	–2.37	–4.02	–4.01	5.05	5.08	–0.86

[a)] This work, phosphate buffer pH 7.0, EDTA, NaCl, 20 °C, ^{31}P NMR reference 85% H_3PO_4.[b)] Ott at al., 25 mM Hepes, pH 8.0, 25 mM EDTA, and 50 mM NaCl, at 19°C (1). [c)] Ott at al., measurement at 32°C, data deducted from original Figure 3 in the Ref. (1). [d)] Wu at al., deducted from original Figure 2 (dashed contours) in the Ref. (2). [e)] Wu at al., deducted from original Figure 2 (solid contours) in the Ref. (2). [f)] Tian at al., 100 mM phosphate buffer, 99% D_2O, 30°C, deducted from original Figure 3 in the Ref.(3).

Table S2. The δ_{31P} shifts (ppm) referenced to A6pT7 phosphate, as measured in the DNA.

DNA step	a)	b)	c)	d)	e)	f)	$\Delta\delta_{31P}$ g)
C1pG2	0.40	0.36	0.35	0.37	0.34	0.33	0.07
G2pC3	0.28	0.26	0.23	0.24	0.20	0.21	0.08
C3pG4	0.50	0.47	0.40	0.40	0.36	0.38	0.14
G4pA5	0.23	0.22	0.25	0.30	0.26	0.27	0.08
A5pA6	0.12	0.12	0.14	0.13	0.10	0.13	0.04
A6pT7	0.00	0.00	0.00	0.00	0.00	0.00	0.00
T7pT8	0.03	0.03	0.01	0.02	0.03	0.00	0.03
T8pC9	0.12	0.12	0.12	0.15	0.17	0.13	0.05

C9pG10	0.53	0.49	0.47	0.52	0.54	0.48	0.07
G10pC11	0.36	0.36	0.34	0.36	0.38	0.33	0.05
C11pG12	0.55	0.52	0.48	0.48	0.49	0.47	0.08

[a)] This work, phosphate buffer pH 7.0, EDTA, NaCl, 20 °C, ^{31}P NMR reference 85% H_3PO_4.[b)] Ott at al., 25 mM Hepes, pH 8.0, 25 mM EDTA, and 50 mM NaCl, at 19°C (1). [c)] Ott at al., measurement at 32°C, data deducted from original Figure 3 in the Ref. (1). [d)] Wu at al., deducted from original Figure 2 (dashed contours) in the Ref. (2). [e)] Wu at al., deducted from original Figure 2 (solid contours) in the Ref. (2). [f)] Tian at al., 100 mM phosphate buffer, 99% D_2O, 30°C, deducted from original Figure 3 in the Ref.(3). [g)] Variations of measured NMR shifts: $\Delta\delta_{31P} = \delta_{31P,max} - \delta_{31P,min}$.

Table S3. The δ_{31P} shifts (ppm) measured at different temperatures (°C). Phosphate buffer pH 7.0, EDTA, NaCl; ^{31}P NMR reference 85% H_3PO_4.

DNA step	10°C	15°C	20°C	25°C	30°C	35°C
C1pG2	-2.475	-2.5	-2.524	-2.555	-2.57	-2.59
G2pC3	-2.6	-2.62	-2.639	-2.66	-2.68	-2.7
C3pG4	-2.35	-2.38	-2.421	-2.46	-2.48	-2.52
G4pA5	-2.71	-2.7	-2.692	-2.68	-2.68	-2.7
A5pA6	-2.8	-2.795	-2.797	-2.8	-2.82	-2.82
A6pT7	-2.88	-2.9	-2.918	-2.92	-2.93	-2.94
T7pT8	-2.8	-2.84	-2.869	-2.9	-2.91	-2.94
T8pC9	-2.8	-2.795	-2.797	-2.8	-2.82	-2.82
C9pG10	-2.35	-2.38	-2.394	-2.4	-2.42	-2.45
G10pC11	-2.52	-2.535	-2.551	-2.555	-2.57	-2.59
C11pG12	-2.31	-2.34	-2.371	-2.4	-2.42	-2.45

Table S4. The δ_{31P} shifts (ppm) measured at different temperatures (°C) referenced to δ_{31P} shift in A6pT7 phosphate at 20 °C.

DNA step	10	15	20	25	30	35	$\Delta\delta_{31P}$ [a)]
C1pG2	0.443	0.418	0.394	0.363	0.348	0.328	0.115
G2pC3	0.318	0.298	0.279	0.258	0.238	0.218	0.100
C3pG4	0.568	0.538	0.497	0.458	0.438	0.398	0.170
G4pA5	0.208	0.218	0.226	0.238	0.238	0.218	0.030
A5pA6	0.118	0.123	0.121	0.118	0.098	0.098	0.025

A6pT7	0.038	0.018	0	-0.002	-0.012	-0.022	0.060
T7pT8	0.118	0.078	0.049	0.018	0.008	-0.022	0.140
T8pC9	0.118	0.123	0.121	0.118	0.098	0.098	0.025
C9pG10	0.568	0.538	0.524	0.518	0.498	0.468	0.100
G10pC11	0.398	0.383	0.367	0.363	0.348	0.328	0.070
C11pG12	0.608	0.578	0.547	0.518	0.498	0.468	0.140

[a)] Variation of δ_{31P} shift due to temperature calculated as maximal δ_{31P} shift minus minimal δ_{31P} shift.

Table S5. The $^{3}J_{P,H3'}$ couplings (Hz) measured in the DNA.

DNA step	Clore [a]	Sklenar [b]		Wu [c]	Average [d]
C1pG2	6.4±0.06	6.3	6.4	5.96±0.04	6.27
G2pC3	3.6±0.2	3.8	3.4	3.76±0.03	3.64
C3pG4	5.7±0.2	5.5	5.8	5.30±0.04	5.58
G4pA5	4.0±0	4	4	4.01±0.03	4.00
A5pA6	2.9±0.4	3.3	2.6	3.01±0.06	2.95
A6pT7	2.6±0.4	2.8	2.3	2.43±0.08	2.53
T7pT8	2.7±0.4	3	2.3	2.74±0.08	2.68
T8pC9	3.0±0.4	3.4	2.6	3.29±0.06	3.07
C9pG10	5.0±0.06	5	5.1	5.16±0.04	5.07
G10pC11	4.1±0.2	3.9	4.2	3.96±0.03	4.04
C11pG12	5.2±0.06	5.2	5.1	5.32±0.04	5.21

[a] Clore et al..(4), [b] Sklenar and Bax, experiments I and II in the Ref.(5), [c] Wu et al..(2) [d] Average value of the experimental $^{3}J_{P,H3'}$ coupling values.

Table S6. The backbone torsion angles (degrees), σ_{31P} shielding (ppm), δ_{31P} shift (ppm) referenced to σ_{31P} in BB00 phosphate, $^{3}J_{P,H3'}$ coupling (Hz) and average population weight *w* in DNA (percent) with the bsc1 and OL21 force fields in NtC phosphates.

NtC	$\delta_{5'}$	ε	ζ	α	β	γ	$\delta_{3'}$	σ_{31P}	δ_{31P}	$^{3}J_{P,H3'}$	*w* (bsc1)	*w* (OL21)
BB00	138	183	258	304	180	44	138	292.19	0	1.92	30.11	33.47
BB07	144	247	169	297	141	46	141	287.96	4.23	10.86	16.42	17.94
BB01	131	181	266	301	176	49	120	292.33	–0.14	1.46	19.54	16.37
BB04	140	201	214	315	153	46	140	291.55	0.64	5.81	4.98	6.41

BA05	131	184	269	296	169	52	104	292.25	–0.06	1.95	5.19	5.82
AB01	86	186	281	301	179	54	142	292.02	0.17	2.52	4.40	5.13
IC05	141	255	174	289	178	50	147	288.56	3.63	10.15	2.12	2.98
BA17	149	253	177	295	131	44	98	287.50	4.69	10.05	1.02	2.33
BA01	136	189	255	300	161	53	88	291.67	0.52	3.07	1.98	2.08
BB16	138	221	282	284	173	48	140	291.64	0.55	10.87	8.22	2.03
AB03	103	195	255	321	162	40	136	292.21	–0.02	4.23	0.71	1.92
BA08	139	208	213	301	141	49	89	289.46	2.73	7.67	0.29	0.59
BB08	147	249	181	66	225	209	148	287.79	4.39	10.83	0.15	0.52
BB17	129	145	275	230	241	79	136	291.86	0.33	3.02	0.09	0.49
BB03	145	175	274	163	166	175	146	289.65	2.54	0.62	0.57	0.25
OP22	147	245	126	286	162	49	142	290.32	1.87	11.00	0.42	0.24
AA02	88	202	274	293	161	54	88	291.19	1.00	6.46	0.07	0.19
AB05	83	214	287	303	176	58	145	291.60	0.59	9.39	0.56	0.14
IC06	137	236	280	288	174	47	142	291.53	0.66	12.35	0.21	0.14
OP19	145	225	64	74	186	188	126	291.53	0.66	11.67	0.00	0.11
AB02	94	59	56	208	188	66	131	288.83	3.36	38.45	0.01	0.08
BA16	146	246	190	61	229	199	85	287.60	4.59	11.23	0.01	0.05
ZZ02	144	269	78	229	175	55	86	289.87	2.32	7.50	0.00	0.04
BB11	145	199	200	123	227	187	144	288.47	3.71	5.65	0.00	0.03
AB04	87	215	297	280	209	55	139	290.88	1.30	10.08	0.04	0.03
BB12	140	196	280	257	76	171	140	290.74	1.45	5.04	2.31	0.02
BB05	142	220	197	76	233	213	129	287.32	4.87	10.55	0.01	0.02
BB20	143	294	110	150	199	54	152	288.42	3.77	1.28	0.00	0.02
IC01	83	220	290	297	223	54	145	290.45	1.74	10.71	0.00	0.01
BB15	144	189	257	345	189	350	148	291.82	0.37	3.01	0.00	0.01
BB14	110	104	305	220	255	83	133	292.77	–0.58	22.08	0.00	0.01
AA09	87	232	272	302	154	52	85	290.79	1.40	12.00	0.00	0.01
BA13	142	220	200	81	231	196	88	287.71	4.48	10.45	0.01	0.01
OP24	148	284	96	82	248	190	85	290.87	1.32	3.51	0.00	0.01
AA00	82	206	288	293	173	55	82	291.54	0.65	7.47	0.02	0.01
OP28	82	226	166	292	159	43	145	289.20	2.99	10.84	0.00	0.00
BB02	141	194	246	31	195	297	150	292.58	–0.39	3.75	0.02	0.00
BA10	136	200	236	95	219	205	90	289.73	2.46	5.57	0.00	0.00
IC04	85	206	287	194	181	183	149	288.84	3.35	7.70	0.00	0.00
OP09	82	198	269	203	146	51	148	288.78	3.41	5.78	0.00	0.00
BA09	134	200	287	256	68	172	90	291.28	0.91	5.74	0.06	0.00
BB13	143	187	293	219	98	161	146	288.05	4.14	2.50	0.06	0.00

Table S7. The NtC classification of DNA phosphates in experimental x-ray structures.

Chain A	1BNA	4BNA	4C64	436D	463D	1FQ2	1EHV
C1pG2	BB04	BB00	BB00	BB00	n.a.	BB00	OP21
G2pC3	BA05	BA01	BA01	BA01	BA01	BA08	BA05

C3pG4	AB01	AB01	AB01	AB01	AB01	AB01	AB01
G4pA5	BB04	BB00	BB00	BB00	BB00	BB00	BB00
A5pA6	BB01	BB00	BB01	BB01	BB00	BB01	BB00
A6pT7	BA05	BA05	BA05	BA05	BB01	BA05	BB01
T7pT8	BB01	BA05	BB01	BB01	BB01	BB01	AB03
T8pC9	BB01	AB01	BB01	BB00	BB01	BB01	BB00
C9pG10	BB00	BB00	BB00	BB00	AB03	BB00	BB00
G10pC11	BB07	BB07	BB07	BB07	BB07	BB07	BB07
C11pG12	BB01	BB01	BA05	BA05	IC06	BB15	IC06
Chain B	1BNA	4BNA	4C64	436D	463D	1FQ2	1EHV
C1pG2	BB00	BB07	BB04	BB07	n.a.	BA05	n.a.
G2pC3	BA05	BA08	BA05	BA05	BB00	BA05	n.a.
C3pG4	AB01	AB01	AB01	AB01	BB00	AB01	n.a.
G4pA5	BB00	BB04	BB00	BB00	BB00	BB00	n.a.
A5pA6	BB00	BB01	BB01	BB01	BB01	BA05	n.a.
A6pT7	BB01	BA05	BA05	BA05	BB01	BB01	n.a.
T7pT8	BB01	AA02	BB01	BB01	BB01	BB01	n.a.
T8pC9	BB01	BA05	BB01	BB01	BA05	BA05	n.a.
C9pG10	BB00	AB01	BB00	BB00	AB01	AB01	n.a.
G10pC11	BA17	BA17	BA08	BB07	BB07	BA08	n.a.
C11pG12	BA05	AA04	AA00	BA01	IC06	AA00	n.a.

Table S8. The NtC classification of DNA phosphates in experimental NMR structures.

Chain A	1NAJ-1	1NAJ-2	1NAJ-3	1NAJ-4	1NAJ-5	1DUF-1	1DUF-2	1DUF-3	1DUF-4	1DUF-5	2DAU	1GIP-1	1GIP-2
C1pG2	BB00	BB00	BB00	BB00	BB00	BB00	BB00	BB00	BB00	BB00	BB00	BB00	BB00
G2pC3	BB00	BB01	BB00	BB01	BB00	BB01	BB01	BB01	BB01	BB01	BB00	BB01	BB01
C3pG4	BB00	BB00	BB00	BB00	BB00	BB00	BB00	BB01	BB00	BB00	BB00	BB00	BB00
G4pA5	BB00	BB00	BB00	BB00	BB00	BB00	BB00	BB00	BB00	BB00	BB00	BB00	BB00
A5pA6	BB01	BB00	BB00	BB00	BB00	BB00	BB00	BB00	BB00	BB00	BB00	BB00	BB00
A6pT7	BA05	BA05	BA05	BA05	BA05	BB01	BB01	BB01	BB01	BB01	BA05	BB01	BB01
T7pT8	BB01	BB01	BB01	BB01	BB01	BB01	BB01	BB01	BB01	BB01	BB01	BB00	BB01
T8pC9	BB00	BB00	BB00	BB00	BB00	BB01	BB01	BB01	BB01	BB01	BB00	BB01	BB01
C9pG10	BB00	BB00	BB00	BB00	BB00	BB00	BB00	BB00	BB00	BB00	BB00	BB00	BB00
G10pC11	BB01	BB01	BB01	BB01	BB01	BA05	BB01	BB01	BA05	BA05	BB00	BB01	BB01
C11pG12	BB01	BB01	BB01	BB01	BB01	BB01	BB01	BB01	BB01	BB01	BB00	BB01	BB01
Chain B	1NAJ-1	1NAJ-2	1NAJ-3	1NAJ-4	1NAJ-5	1DUF-1	1DUF-2	1DUF-3	1DUF-4	1DUF-5	2DAU	1GIP-1	1GIP-2
C1pG2	BB00	BB00	BB00	BB00	BB00	BB00	BB00	BB00	BB00	BB00	BB00	BB00	BB00
G2pC3	BB00	BB00	BB00	BB00	BB00	BB01	BB01	BB01	BB01	BB01	BB00	BB01	BB01
C3pG4	BB00	BB00	BB00	BB00	BB00	BB00	BB00	BB01	BB00	BB00	BB00	BB00	BB00
G4pA5	BB00	BB00	BB00	BB00	BB00	BB00	BB00	BB00	BB00	BB00	BB00	BB00	BB00
A5pA6	BB01	BB01	BB00	BB00	BB00	BB00	BB00	BB00	BB00	BB00	BB00	BB00	BB00
A6pT7	BA05	BA05	BA05	BA05	BA05	BB01	BB01	BB01	BB01	BB01	BA05	BB01	BB01
T7pT8	BB01	BB01	BB01	BB01	BB01	BB01	BB01	BB01	BB01	BB01	BB01	BB00	BB01
T8pC9	BB00	BB00	BB00	BB00	BB00	BB01	BB01	BB01	BB01	BB01	BB00	BB01	BB01
C9pG10	BB00	BB00	BB00	BB00	BB00	BB00	BB01	BB00	BB00	BB00	BB00	BB00	BB00

G10pC11	BB01	BB01	BB01	BB01	BB01	BA05	BA05	BB01	BB01	BA05	BB00	BB01	BB01
C11pG12	BB01	BB01	BB01	BB01	BB01	BB01	BB01	BB01	BB01	BB01	BB00	BB01	BB01

Table S9A. The δ_{31P} NMR shifts (ppm) assigned in experimental X-ray PDB structures.

DNA	Range	1FQ2	4C64	436D	4BNA	1BNA	Exp. [a]
C1pG2	0.07÷2.18	0.07	0.38	2.18	2.18	0.42	0.37
G2pC3	0.04÷1.69	1.44	0.29	0.29	1.69	0.04	0.26
C3pG4	0.23÷0.27	0.27	0.23	0.23	0.23	0.27	0.46
G4pA5	0.06÷0.42	0.10	0.06	0.06	0.38	0.42	0.24
A5pA6	−0.08÷0.03	0.00	−0.08	−0.08	−8.2E-3	0.03	0.12
A6pT7	0.00	0.00	0.00	0.00	0.00	0.00	0.00
T7pT8	−0.08÷0.53	−0.04	−0.08	−0.08	0.53	−0.04	0.02
T8pC9	−0.08÷0.11	0.00	−0.08	−8.2E-3	0.11	−0.04	0.12
C9pG10	0.06÷0.19	0.19	0.06	0.06	0.15	0.10	0.52
G10pC11	3.54÷4.56	3.58	3.54	4.29	4.52	4.56	0.37
C11pG12	0.00÷1.19	0.61	0.36	0.29	1.19	0.00	0.52
MAD [b]	0.99÷1.47	1.05	0.98	1.32	1.46	1.29	-

[a] Measured in phosphate buffer, pH 7.0, EDTA, NaCl, 20 °C. [b] Mean absolute deviation.

Table S9B. The δ_{31P} NMR shifts (ppm) assigned in experimental NMR PDB structures.

DNA	Range	1NAJ	2DAU	1DUF	1GIP	Exp. [a]
C1pG2	0.06÷0.14	0.06	0.06	0.14	0.14	0.37
G2pC3	0.00÷0.06	0.04	0.06	0.00	0.00	0.26
C3pG4	0.06÷0.14	0.06	0.06	0.13	0.14	0.46
G4pA5	0.06÷0.14	0.06	0.06	0.14	0.14	0.24
A5pA6	0.02÷0.14	0.02	0.06	0.14	0.14	0.12
A6pT7	0.00	0.00	0.00	0.00	0.00	0.00
T7pT8	−0.08÷0.07	−0.08	−0.08	0.00	0.07	0.02
T8pC9	0.00÷0.06	0.06	0.06	0.00	0.00	0.12
C9pG10	0.06÷0.14	0.06	0.06	0.13	0.14	0.52
G10pC11	−0.08÷0.06	−0.08	0.06	0.05	0.00	0.37
C11pG12	−0.08÷0.06	−0.08	0.06	0.00	0.00	0.52

MAD [b]	0.32÷0.24	0.32	0.27	0.26	0.26	-

[a] Measured in phosphate buffer, pH 7.0, EDTA, NaCl, 20 °C. [b] Mean absolute deviation.

Table S10A. The $^{3}J_{P,H3'}$ coupling constants (Hz) assigned in experimental crystal PDB structures.

DNA	Range	1FQ2	4C64	436D	4BNA	1BNA	Exp. [a]
C1pG2	1.94÷6.39	1.94	3.86	6.39	6.39	3.86	6.27
G2pC3	1.95÷5.37	4.81	2.51	2.51	5.37	1.95	3.64
C3pG4	2.52	2.52	2.52	2.52	2.52	2.52	5.58
G4pA5	1.92÷3.86	1.92	1.92	1.92	3.86	3.86	4.00
A5pA6	1.45÷1.70	1.70	1.45	1.45	1.68	1.68	2.95
A6pT7	1.70÷1.95	1.70	1.95	1.95	1.95	1.70	2.53
T7pT8	1.45÷4.20	1.45	1.45	1.46	4.20	1.45	2.69
T8pC9	1.45÷2.23	1.70	1.45	1.68	2.23	1.45	3.07
C9pG10	1.92÷2.22	2.22	1.92	1.92	2.22	1.92	5.07
G10pC11	9.26÷10.86	9.26	9.26	10.86	10.45	10.45	4.04
C11pG12	1.70÷5.24	5.24	4.71	2.51	4.03	1.70	5.21
MAD [b]	2.54÷2.46	2.61	2.43	2.77	2.49	2.82	-

[a] Average $^{3}J_{P,H3'}$ coupling in the four experiments (Table S5). [b] Mean absolute deviation.

Table S10B. The $^{3}J_{P,H3'}$ coupling constants (Hz) assigned in experimental NMR PDB structures.

DNA	Range	1NAJ	2DAU	1DUF	1GIP	Exp. [a]
C1pG2	1.92	1.92	1.92	1.92	1.92	6.27
G2pC3	1.45÷1.92	1.83	1.92	1.45	1.45	3.64
C3pG4	1.87÷1.92	1.92	1.92	1.87	1.92	5.58
G4pA5	1.92	1.92	1.92	1.92	1.92	4.00
A5pA6	1.78÷1.92	1.78	1.92	1.92	1.92	2.95
A6pT7	1.45÷1.95	1.95	1.95	1.45	1.45	2.53
T7pT8	1.45÷1.68	1.45	1.45	1.45	1.68	2.69
T8pC9	1.45÷1.92	1.92	1.92	1.87	1.92	3.07
C9pG10	1.87÷1.92	1.92	1.92	1.87	1.92	5.07

G10pC11	1.45÷1.92	1.45	1.92	1.75	1.45	4.04
C11pG12	1.45÷1.92	1.45	1.92	1.45	1.45	5.21
MAD [b]	2.50÷2.32	2.61	2.50	2.66	2.66	-

[a] Average $^3J_{P,H3'}$ coupling in the four experiments (Table S5). [b] Mean absolute deviation.

Table S11. The σ_{31P} shielding (ppm), δ_{31P} shift (ppm) and $^3J_{PH3'}$ coupling (Hz) calculated in four solvated NtC phosphates.

Parameter	Solvation	BB00	BB04	BB07	BB01
σ_{31P}	-	292.19	291.55	287.96	292.33
σ_{31P}	Mg^{2+}	293.00	291.45	290.45	293.00
δ_{31P} [a]	-	0	0.64	4.23	−0.14
δ_{31P} [b]	Mg^{2+}	0	1.55	2.55	0.00
$^3J_{PH3'}$	-	1.92	5.81	10.86	1.45
$^3J_{PH3'}$	Mg^{2+}	1.81	5.65	11.07	1.27
$\Delta\sigma_{31P}$ [c]	-	0.81	−0.10	2.49	0.66
$\Delta^3J_{PH3'}$ [d]	-	−0.11	−0.16	0.21	−0.18

[a] Referenced to σ_{31P} in hydrated BB00 phosphate. [b] Referenced to σ_{31P} in solvated BB00 phosphate. [c] $\Delta\sigma_{31P} = \sigma_{31P}$ (Mg^{2+}) – σ_{31P} (NO Mg^{2+}). [d] $\Delta^3J_{PH3'} = {}^3J_{PH3'}$ (Mg^{2+}) – $^3J_{PH3'}$ (NO Mg^{2+}).

Table S12. The NtC weights calculated and fitted to $^3J_{P,H3'}$ experiment.

DNA	MD [a]				Fitted [b]				Fitted plus Mg^{2+} solvation [a]			
	BB00	BB01	BB07	BB04	BB00	BB01	BB07	BB04	BB00	BB01	BB07	BB04
C1pG2	20.17	4.01	35.61	14.04	32.05	6.37	25.39	10.01	31.96	6.35	25.47	10.04
G2pC3	49.39	15.53	10.41	4.47	50.89	16.00	9.03	3.88	50.19	15.78	9.67	4.16
C3pG4	44.64	5.96	18.27	7.09	37.02	4.84	24.29	9.61	37.19	4.97	24.35	9.46
G4pA5	20.71	5.01	51.78	8.40	56.74	13.74	13.27	2.15	56.16	13.60	13.89	2.25
A5pA6	47.22	18.32	10.98	7.43	53.72	20.85	5.60	3.78	52.82	20.50	6.35	4.29
A6pT7	20.04	44.33	0.40	0.88	17.34	38.36	3.11	6.85	16.86	37.30	3.59	7.91
T7pT8	24.60	36.73	0.29	1.58	21.00	31.35	1.66	9.18	20.28	30.28	1.94	10.70
T8pC9	46.71	18.82	3.12	2.78	43.06	18.08	5.57	4.97	44.03	17.73	5.12	4.56
C9pG10	28.67	6.45	29.16	11.71	39.58	8.90	19.63	7.88	39.27	8.83	19.91	7.99
G10pC11	41.76	12.15	20.07	4.38	50.62	14.73	10.68	2.33	50.04	14.56	11.29	2.46
C11pG12	26.20	13.66	18.27	8.10	26.54	13.85	17.90	7.94	26.30	13.73	18.15	8.05

[a] Calculated with the OL21 force field. [b] Fitted to $^3J_{P,H3'}$ experiment. [c] Fitted with solvated NtC phosphates.

Table S13. The NtC weights calculated and fitted to δ_{31P} experiment.

DNA	MD [a]				Fitted [b]				Fitted plus Mg^{2+} solvation [a]			
	BB00	BB01	BB07	BB04	BB00	BB01	BB07	BB04	BB00	BB01	BB07	BB04
C1pG2	20.17	4.01	35.61	14.04	61.26	12.17	0.28	0.11	55.83	11.09	4.95	1.95

G2pC3	49.39	15.53	10.41	4.47	48.06	15.52	11.49	5.08	48.90	15.37	10.86	4.67
C3pG4	44.64	5.96	18.27	7.09	55.43	7.41	9.45	3.67	44.02	5.88	18.77	4.97
G4pA5	20.71	5.01	51.78	8.40	67.35	16.30	1.93	0.31	58.48	14.16	11.42	1.85
A5pA6	47.22	18.32	10.98	7.43	48.71	18.28	10.53	6.88	48.03	18.63	10.31	6.98
A6pT7 [d]	20.04	44.33	0.40	0.88	17.34	38.36	3.11	6.85	16.86	37.30	3.59	7.91
T7pT8	24.60	36.73	0.29	1.58	20.05	29.94	2.02	11.19	20.92	31.23	1.69	9.36
T8pC9	46.71	18.82	3.12	2.78	45.01	18.12	4.39	3.91	39.73	16.00	8.31	7.40
C9pG10	28.67	6.45	29.16	11.71	53.51	12.03	7.47	3.00	44.13	9.92	15.66	6.29
G10pC11	41.76	12.15	20.07	4.38	58.05	16.89	2.99	0.67	51.01	14.84	10.27	2.24
C11pG12	26.20	13.66	18.27	8.10	36.81	19.20	7.08	3.14	32.06	16.72	12.09	5.36

[a] Calculated with the OL21 force field. [b] Fitted to δ_{31P} experiment. [c] Fitted with solvated NtC phosphates. [d] The reference phosphate, the weights fitted to $^{3}J_{P,H3'}$ experiment.

Table S14. The dependence of δ_{31P} shifts (ppm) on temperature calculated by Population weighting with OL21 force field.

DNA Step	5 °C	10 °C	15 °C	20 °C	25 °C	30 °C	35 °C	40 °C	Max	Min	Variation
C1pG2	2.22	2.19	2.16	2.10	2.07	2.02	1.99	1.96	2.22	1.96	0.26
G2pC3	0.70	0.67	0.68	0.67	0.73	0.72	0.76	0.75	0.76	0.67	0.09
C3pG4	1.01	0.90	0.94	0.96	0.91	1.03	0.98	1.02	1.03	0.90	0.13
G4pA5	2.71	2.82	2.74	2.63	2.71	2.51	2.51	2.43	2.82	2.43	0.39
A5pA6	0.57	0.64	0.62	0.64	0.64	0.65	0.67	0.68	0.68	0.57	0.11
A6pT7	-0.05	0.00	0.02	0.00	-0.01	0.00	0.01	0.05	0.05	-0.05	0.10
T7pT8	-0.01	0.00	0.03	0.03	0.05	0.04	0.05	0.06	0.06	-0.01	0.07
T8pC9	0.24	0.17	0.19	0.14	0.20	0.24	0.24	0.32	0.32	0.14	0.18
C9pG10	1.65	1.81	1.72	1.75	1.70	1.59	1.61	1.56	1.75	1.56	0.19
G10pC11	1.34	1.29	1.33	1.30	1.35	1.33	1.33	1.33	1.35	1.29	0.06
C11pG12	1.18	1.12	1.19	1.14	1.14	1.19	1.13	1.16	1.19	1.12	0.07

Table S15. The dependence of δ_{31P} shifts (ppm) on temperature calculated by Probability averaging with OL21 force field.

DNA Step	5 °C	10 °C	15 °C	20 °C	25 °C	30 °C	35 °C	40 °C	Max	Min	Variation
C1pG2	2.48	2.44	2.41	2.34	2.30	2.24	2.20	2.17	2.48	2.17	0.31
G2pC3	0.69	0.67	0.67	0.67	0.74	0.72	0.76	0.75	0.76	0.67	0.09
C3pG4	1.04	0.92	0.98	1.00	0.94	1.09	1.03	1.08	1.09	0.92	0.17
G4pA5	2.86	2.97	2.89	2.76	2.84	2.64	2.62	2.53	2.97	2.53	0.44
A5pA6	0.65	0.73	0.70	0.72	0.73	0.73	0.76	0.77	0.77	0.65	0.12
A6pT7	-0.05	0.00	0.01	0.00	-0.01	0.00	0.02	0.05	0.05	-0.05	0.10
T7pT8	0.01	0.01	0.04	0.03	0.06	0.05	0.06	0.07	0.07	0.01	0.06
T8pC9	0.19	0.12	0.14	0.10	0.16	0.20	0.21	0.30	0.30	0.10	0.20
C9pG10	1.83	2.01	1.90	1.94	1.89	1.75	1.79	1.72	2.01	1.72	0.29
G10pC11	1.31	1.27	1.31	1.28	1.33	1.31	1.32	1.31	1.33	1.27	0.06
C11pG12	1.31	1.24	1.31	1.27	1.26	1.31	1.25	1.28	1.31	1.24	0.07

Table S16. The solely effect on δ_{31P} shifts due to Probability averaging ($\Delta\delta_{31P}$) at different temperatures calculated with OL21 force fiel, $\Delta\delta_{31P}$ = δ_{31P} (Population weighting) – δ_{31P} (Probability averaging).

DNA Step	5 °C	10 °C	15 °C	20 °C	25 °C	30 °C	35 °C	40 °C	Max	Min	Variation

C1pG2	0.26	0.26	0.25	0.24	0.23	0.22	0.22	0.21	0.26	0.21	0.05
G2pC3	-0.01	-0.01	0.00	-0.01	0.00	0.00	0.00	0.00	0.00	-0.01	0.01
C3pG4	0.04	0.02	0.04	0.04	0.03	0.05	0.05	0.06	0.06	0.02	0.04
G4pA5	0.15	0.15	0.15	0.13	0.13	0.12	0.11	0.10	0.15	0.10	0.05
A5pA6	0.07	0.08	0.08	0.08	0.09	0.08	0.09	0.09	0.09	0.07	0.02
A6pT7	-0.01	0.00	0.00	0.00	0.00	0.00	0.00	0.00	0.00	-0.01	0.01
T7pT8	0.01	0.01	0.01	0.01	0.01	0.00	0.01	0.01	0.01	0.01	0.01
T8pC9	-0.05	-0.05	-0.04	-0.04	-0.03	-0.03	-0.02	-0.02	-0.02	-0.05	0.02
C9pG10	0.18	0.19	0.18	0.19	0.18	0.16	0.17	0.16	0.19	0.16	0.03
G10pC11	-0.02	-0.01	-0.01	-0.02	-0.01	-0.01	-0.01	-0.01	-0.01	-0.02	0.01
C11pG12	0.13	0.12	0.12	0.12	0.12	0.12	0.12	0.12	0.13	0.12	0.01

Supplementary References

1. Ott, J. and Eckstein, F. (1985) P-31 Nmr Spectral-Analysis of the Dodecamer D(Cgcgaattcgcg). *Biochemistry-Us*, **24**, 2530-2535.
2. Wu, Z.R., Tjandra, N. and Bax, A. (2001) Measurement of (1)H3 '-P-31 dipolar couplings in a DNA oligonucleotide by constant-time NOESY difference spectroscopy. *Journal of Biomolecular Nmr*, **19**, 367-370.
3. Tian, Y., Kayatta, M., Shultis, K., Gonzalez, A., Mueller, L.J. and Hatcher, M.E. (2009) NMR Investigation of Backbone Dynamics in DNA Binding Sites. *J Phys Chem B*, **113**, 2596-2603.
4. Clore, G.M., Murphy, E.C., Gronenborn, A.M. and Bax, A. (1998) Determination of three-bond (1)H3 '-P-31 couplings in nucleic acids and protein nucleic acid complexes by quantitative J correlation spectroscopy. *Journal of Magnetic Resonance*, **134**, 164-167.
5. Sklenar, V. and Bax, A. (1987) MEASUREMENT OF H-1-P-31 NMR COUPLING-CONSTANTS IN DOUBLE-STRANDED DNA FRAGMENTS. *Journal of the American Chemical Society*, **109**, 7525-7526.
